# Multi-loaded PLGA microspheres as neuroretinal therapy in a chronic glaucoma animal model

**DOI:** 10.1007/s13346-024-01702-x

**Published:** 2024-10-03

**Authors:** Alba Aragón-Navas, Maria Jesus Rodrigo, Inés Munuera, David García-Herranz, Manuel Subías, Pilar Villacampa, Julián García-Feijoo, Luis Pablo, Elena Garcia-Martin, Rocio Herrero-Vanrell, Irene Bravo-Osuna

**Affiliations:** 1 Innovation, Therapy and Pharmaceutical Development in Ophthalmology (InnOftal) Research Group, UCM 920415, Department of Pharmaceutics and Food Technology, Faculty of Pharmacy, Complutense University of Madrid, Madrid, Spain; 2 Health Research Institute, San Carlos Clinical Hospital (IdISSC), Madrid, Spain; 3 National Ocular Research Network RD21/0002/0050. RICORS Red de Enfermedades Inflamatorias (RD21/0002), Carlos III Health Institute, Madrid, Spain; 4 Department of Ophthalmology, Miguel Servet University Hospital, Zaragoza, Spain; 5 Miguel Servet Ophthalmology Research Group (GIMSO), Aragon Health Research Institute (IIS Aragon), University of Zaragoza, Zaragoza, Spain; 6 Biotech Vision, Instituto Oftalmologico Quiron, Zaragoza, Spain; 7 Department of Physiological Sciences, Faculty of Medicine and Health Sciences, University of Barcelona and Bellvitge Biomedical Research Institute (IDIBELL), Feixa Llarga S/N, 08907 L’Hospitalet de Llobregat, Spain; 8 Department of Ophthalmology, San Carlos Clinical Hospital, Health Research Institute of the San Carlos Clinical Hospital (IdISSC), Madrid, Spain; 9 University Institute for Industrial Pharmacy (IUFI), School of Pharmacy, Complutense University of Madrid, Madrid, Spain; 10 National Ocular Pathology Network (OFTARED), Carlos III Health Institute, Madrid, Spain

**Keywords:** Co-delivery, Neuroprotection, Poly lactic-co-glycolic acid (PLGA), Microspheres, Retinal neurodegenerative diseases, Glaucoma animal model

## Abstract

This work focused on the co-encapsulation and simultaneous co-delivery of three different neuroprotective drugs in PLGA (poly(lactic-co-glycolic acid) microspheres for the treatment of glaucoma. For formulation optimization, dexamethasone (anti-inflammatory) and ursodeoxycholic acid (anti-apoptotic) were co-loaded by the solid-in-oil-in-water emulsion solvent extraction-evaporation technique as a first step. The incorporation of a water-soluble co-solvent (ethanol) and different amounts of dexamethasone resulted critical for the encapsulation of the neuroprotective agents and their initial release. The optimized formulation was obtained with 60 mg of dexamethasone and using an 80:20 dichloromethane:ethanol ratio. In the second step in the microencapsulation process, the incorporation of the glial cell line-derived neurotrophic factor (GDNF) was performed. The final prototype showed encapsulation efficiencies for each component above 50% with suitable properties for long-term application for at least 3 months. Physicochemical studies were performed by SEM, TEM, DSC, XRD, and gas chromatography. The evaluation of the kinetic release by the Gallagher-Corrigan analysis with Gorrasi correction helped to understand the influence of the co-microencapsulation on the delivery of the different actives from the optimized formulation. The final prototype was tested in a chronic glaucoma animal model. Rats received two intravitreal injections of the neuroprotective treatment within a 24-week follow-up study. The proposed formulation improved retinal ganglion cell (RGC) functionality examined by electroretinography. Also, it was able to maintain a neuroretinal thickness similar to that of healthy animals scanned by in vivo optical coherence tomography, and a higher RGC count on histology compared to glaucomatous animals at the end of the study.

**Graphical Abstract:**

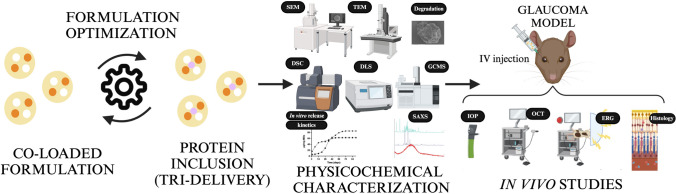

**Supplementary Information:**

The online version contains supplementary material available at 10.1007/s13346-024-01702-x.

## Introduction

Glaucoma is a multifactorial neurodegenerative disorder and one of the leading causes of irreversible vision loss, included in the top three causes of blindness in 2015 [1]. The prevalence has increased over the years, and it is estimated a rise of 74% from 2013 until 2040 [2]. Glaucoma is characterized by the death of retinal ganglion cells (RGC) and the loss of their axons in the optic nerve. Currently, the elevation of intraocular pressure (IOP) is one of the most important risk factors and the only one modifiable [3]. Therefore, most of the pharmacological treatments focus on lowering IOP by different mechanisms: prostaglandin analogues, beta-blockers, alpha agonists, carbonic anhydrase inhibitors, and cholinergic agents [4].

However, despite reducing IOP, visual loss continues progressing in some patients and in many others, glaucomatous retinal and optic nerve degeneration is observed in absence of IOP elevation (normotensive patients). Therefore, new strategies such as neuroprotection have emerged. In the case of glaucoma, this therapeutic approach aims to prevent or improve the survival of RGCs and optic nerve fibers. Consequently, neuroprotection targets RGC protection, being a way to reduce RGCs loss in all cases (IOP-dependent and independent patients) [5–8]. There are different processes responsible for RGC apoptosis: oxidative stress, hyperglycemia, inflammation, glutamate excitotoxicity, ischemia, mitochondrial dysfunction, aggregation of misfolded proteins, neurotrophic deprivation, glial activation, and axonal transport dysregulation [7, 9, 10]. Recently, the role of microglia and astroglia cells (Müller cells and astrocytes) in neuroinflammation processes in glaucoma has been demonstrated [11, 12]. Not only one of these events but several of them likely converge to induce RGC loss. This fact suggests that combined therapy could be required to achieve effectiveness for the neuroprotective treatment of glaucoma [7]. In this sense, our research group has previously developed a multi-delivery microsystem loaded with three neuroprotective substances with anti-inflammatory and antioxidant activities (dexamethasone, coenzyme Q10, and melatonin). This formulation showed beneficial results in RGCs survival in an experimental glaucoma model [13].

It exists, theoretically, four main routes to administrate drugs into the posterior segment of the eye: topical, systemic, intraocular (intracameral, intravitreal, subretinal), and periocular (subconjunctival, sub-Tenon, yuxtascleral, etc.) [14]. Although the most desirable one is the ocular topical administration [15], the drug bioavailability in the posterior segment is generally reduced by multiple factors such as the short ocular contact time or the lacrimal drainage system, among others [16]. The ocular barriers limit the systemic route, requiring large amounts of drugs, and, consequently, significant general side effects are linked. For periocular administration, the main obstacle is drug penetration through the sclera which limits the drug access into the eye [14]. Although periocular or ocular topical administration of drugs in the eye is being evaluated with promising results to treat retinal diseases in some cases, currently, the intravitreal administration of active substances in suitable vehicles (bolus injection) remains the most used in the clinic [17, 18]. In that approach multiple injections are needed to maintain therapeutic concentrations. However, intraocular injections can cause inconvenience to patients such as adverse effects like cataracts, retinal detachment, and hemorrhages, and increase the risk of local side effects just as the number of administrations increases [14]. Furthermore, bolus administration can produce an initial toxic concentration of drugs in the retinal proximity to the injection site causing additional damage [19]. Nevertheless, intraocular drug delivery systems (IODDS) may avoid these concerns. They can release drugs in a sustained manner for a long time with a single administration instead of multiple injections and avoiding initial potential retinal toxicity. Although biodegradable and not biodegradable polymers can compose IODDS, biodegradable materials are preferred as they are eventually disappearing after releasing the drug without the need for surgery [20]. In this sense, biodegradable microsystems are emerging as an interesting tool to treat chronic posterior segment diseases in recent years. Among their benefits, microparticles can deliver drugs for periods from weeks to months, depending on the disease and patients' needs, and can encapsulate different drugs in the same device [21]. Furthermore, the amount of microparticles administrated into the vitreous can be easily modified. For these points, they are considered promising candidates for personalized therapy [22]. Moreover, microparticles can be easily administrated by suspending and injecting them through different needle gauges (between 25 and 32G) [23]. One of the most popular biodegradable polymers used in clinical devices is the poly (lactic-co-glycolic) acid (PLGA), whose use has been approved for ocular administration by the US Food and Drug Administration (FDA) and European Medicines Agency (EMA).

The present study optimizes a multi-loaded MSs formulation including three different substances with anti-inflammatory and neuroprotective effects: dexamethasone (DX), ursodeoxycholic acid (UDCA), and glial cell line-derived neurotrophic factor (GDNF). The corticosteroid DX is already used to treat ocular inflammation and it is already marketed in an intraocular implant (Ozurdex^®^) for the treatment of macular oedema [24, 25]. UDCA is a component of bile acid with antioxidant, anti-apoptotic, and anti-inflammatory properties, which has also shown neuroprotective effects in retinal disorders [26, 27]. Finally, GDNF has been previously tested proving its neuroprotection effect in retinal diseases [28, 29]. The safety and neuroprotective efficacy of poly lactic-co-glycolic acid microspheres individually loaded with the selected agents have been previously studied, both in retinal cell cultures and in retinal explants, as well as after intravitreal administration in different experimental animals [13, 28–34].

In this work, different technological strategies have been evaluated to obtain this tri-delivery system. In addition, as part of the characterization of the microparticles, an exhaustive study has been carried out on the kinetic mechanisms involved in the release of the active agents, and on the influence that co-microencapsulation and subsequent simultaneous delivery of substances with different natures can have on these mechanisms. These studies can help to understand multi-charged systems more deeply and to elaborate them with greater precision.

The neuroprotective activity of the final prototype has been confirmed in a chronic glaucoma model in rats. In this sense, it exists multiple animal models of glaucoma, the so-called “acute models” where the degeneration occurs very fast, and the “chronic models”, where degeneration occurs more progressively. Acute models do not reproduce the reality of these pathologies nor allow testing of the long-term efficacy of formulations. Several chronic degeneration models are based on IOP increase due to the limitation of aqueous humor outflow with repeated interventions, as the case of Morrison model [35]. To avoid frequent interventions new glaucoma animal models based on the intraocular pressure elevation, and subsequently, retinal glaucomatous damage, by injection of loaded and non-loaded biodegradable microparticles in the anterior chamber of the eye have been proposed in the last years [36–39]. In the present work, one of the mentioned models has been selected for neuroprotection activity studies, where glaucomatous damage in the retina was observed over six months after a single intracameral injection of fibronectin (FN) loaded PLGA microspheres, mimicking the progression of the disease in humans [40]. The main advantage of this animal model is that the chronic retinal damage is created by a single injection of FN-loaded particles in the anterior chamber, with no damage of any other ocular structure, furthermore, this model allows to test intravitreal drug delivery systems for long periods.

## Materials and methods

### Materials

PLGA (50:50) polymer (Resomer^®^503) was acquired from Evonik Nutrition & Care GmbH (Darmstadt, Germany). Ursodeoxycholic acid (UDCA) (purity > 99%) was obtained from Alfa Aesar (Haverhill, Massachusetts, USA) and Dexamethasone (DX) (purity > 98%), DL-alpha-tocopherol acetate was provided by Sigma-Aldrich (St. Louis Mo., USA). Polyvinyl alcohol 72000 g/mol (PVA) was purchased from Panreac Química (Castellar del Vallès, Spain). Recombinant human Glial cell line-derived neurotrophic factor (GDNF), and enzyme-linked immunosorbent assays (ELISA) for GDNF quantification were purchased from R&D Systems (Minneapolis, MN, USA).

All organic solvents were HPLC-grade and used as received.

### Microsphere elaboration

The tri-loaded microspheres were prepared following the Solid/Oil/Water (S/O/W) emulsion solvent extraction-evaporation technique.

In an initial step, DX-UDCA MSs were elaborated. 400 mg of PLGA and different amounts of UDCA (ranging 20–60 mg) were dissolved in 0.9 mL methylene chloride or 1.2 mL of a mixture of methylene chloride:ethanol (different proportions were studied: 75:25; 80:20; 85:15). 60 or 80 mg of DX were incorporated into the polymeric dissolution and dispersed by ultrasonication (Ultrasons; J.P. Selecta**,** Barcelona, Spain) in an ice-water bath for 5 min. 40 µL of alpha-tocopherol acetate were added to the O-phase and then, the mixture was sonicated (Sonicator XL; Heat Systems, Inc., Farmingdale, NY, USA) in an ice-water bath for 1 min and emulsified adding 5 mL of PVA MilliQ^®^ water solution (2% w/v) through a homogenizer (Polytron^®^RECO, Kinematica, GmbHT PT3000, Lucerna, Switzerland) for 1 min at 5000 or 8500 rpms.

The obtained emulsion was incorporated into 100 mL of PVA MilliQ^®^ water solution (0.1% w/v) and it was stirred for 3 h to harden the MSs.

**Fig. 1:**
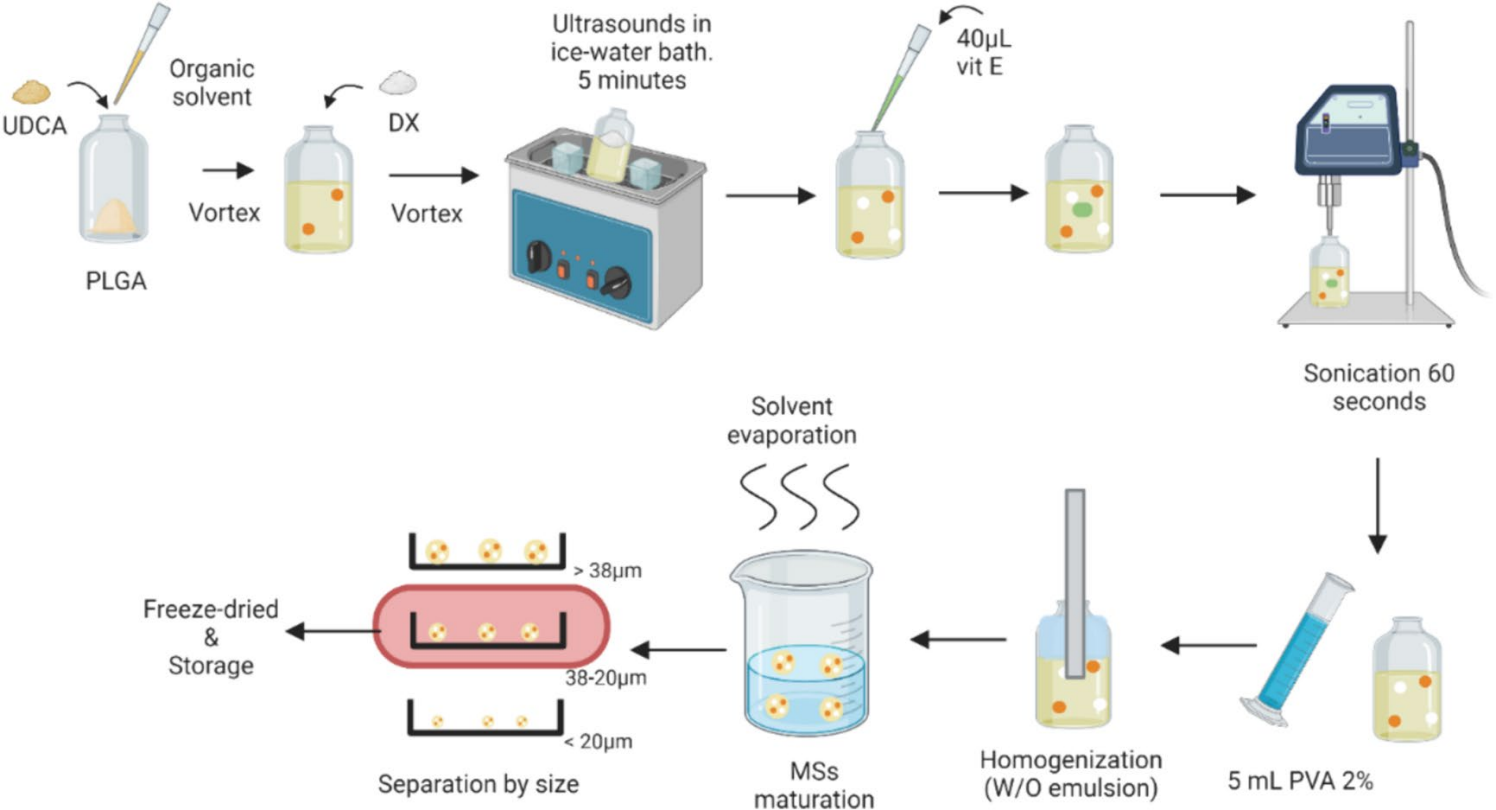
DX-UDCA MSs elaboration. Created with BioRender.com

Once MSs maturated, they were washed and sieved. Finally, MSs were freeze-dried (Freezing: -60 °C /15 min, drying: -60°C/12 h/0.1 mBar) and stored at -30 °C in dry conditions (Fig. 1). Once the formulation was optimized, MSs loaded with only one component (UDCA or DX) were also elaborated at the selected conditions for comparison purposes.

The different conditions studied for the optimisation of the formulation are summarized in Table 1.

**Table 1:** Conditions studied for the formulation optimisation (A-H)

Formulation	Composition (UDCA/DX) (mg/mg)	rpms	Volume of Organic Solvent
A	20/80	5000	0.9 mL MC
B	40/80	5000	0.9 mL MC
C	40/80	8500	1.2 mL MC:EtOH (75:25)
D	60/80	8500	1.2 mL MC:EtOH (75:25)
E	40/80	8500	1.2 mL MC:EtOH (80:20)
F	40/60	8500	1.2 mL MC:EtOH (80:20)
G	40/80	8500	1.2 mL MC:EtOH (85:15)
H	40/60	8500	1.2 mL MC:EtOH (85:15)

In the second step of the optimization process, the third component, the protein, was included in the inner phase of the emulsion. Briefly, 20 µg of GDNF (final formulation) were added to the 40 µL of alpha-tocopherol acetate by using an ultrasonic probe for 30 s. Then, the procedure was carried out as same as already mentioned before (Table 2).

**Table 2:** Summarized conditions of the selected DX-UDCA-MSs formulation (corresponding to F formulation in the formulation optimization process), single-loaded formulations (UDCA-MSs and DX-MSs), and the incorporation of the GDNF in the optimized formulation (DX-UDCA-GDNF-MSs)

Formulation	Composition (UDCA/DX/protein) (mg/mg/µg)	rpms	Volume of Organic Solvent
DX-UDCA-MSs	40/60/-	8500	1.2 mL MC:EtOH (80:20)
UDCA-MSs	40/-/-	8500	1.2 mL MC:EtOH (80:20)
DX-MSs	-/60/-	8500	1.2 mL MC:EtOH (80:20)
DX-UDCA-GDNF-MSs	40/60/20	8500	1.2 mL MC:EtOH (80:20)

### UDCA quantification by LC/MS

The chromatographic system was made of a High-Performance Liquid Chromatography (Waters 1525 binary HPLC pump and Waters 2707 autosampler) coupled to a mass detector (Waters 3100 single quadrupole mass spectrometer). The quantification was performed by employing a Nova-Pak C18 column (4 μm, ID 2.1 mm × 150 mm) connected previously to a guard column (4 μm, 3.9 mm × 20 mm), both were maintained at 45 °C. The mobile phase was composed of acetonitrile/ammonium acetate in water 15 mM (2:1 v/v), adjusted to pH 5.0 with formic acid and the flow rate was 0.15 mL/min.

Electrospray ion source (ESI) operated in a negative ionization mode. The parameters were set as follows: 3 kV for spray voltage, 80 °C for the capillary temperature, and 3 V extractor voltage. Desolvation (925 L/h flow rate, 180 °C desolvation temperature) was performed employing nitrogen gas (> 99.999%).

### DX quantification by LC/MS

The chromatographic system was the same as for UDCA quantification by LC/MS. The mobile phase was 15 mM ammonium acetate in water and acetonitrile (1:1 v/v) and the rate employed was 0.30 mL/min. The ESI source was adjusted to positive ion mode. The instrument settings 3 kV electrospray voltage, nebulization (150 L/h flow rate, 120 °C source temperature, 3 V extractor voltage), and desolvation (500 L/h flow rate, 350 °C desolvation temperature) were performed employing nitrogen gas (> 99.999%).

### Microsphere physicochemical characterization

#### Mean particle size and particle size distribution

The mean particle size and particle size distribution of each formulation were measured by dual light scattering in a Microtrac^®^S3500 Series Particle Size Analyser (Montgomeryville, PA, USA) by suspending MSs in MilliQ^®^ water. The particle size results are expressed as the mean ± standard deviation (SD) (n = 3).

#### Morphological evaluation by scanning electron microscopy (SEM) and transmission electron microscopy (TEM)

The external morphology of MSs was observed by scanning electron microscopy (Jeol, JSM-6335F, Tokyo, Japan). Prior to observation, freeze-dried MSs were applied a gold sputter-coating.

The internal morphology was studied by transmission electron microscopy (TEM, Jeol JEM 1010, Tokyo, Japan). Previously, 50–70 µm of thickness cross-sectional cuts were made out by Reichert Ultracut S Ultramicrotome (Leica Microsystems Inc, Wetzlar, Germany) in samples deposited in a synthetic resin medium (Spurr Low Viscosity Embedding Kit).

Both external and internal morphology were considered key points to the optimization process since the formulations that contained external crystals were discarded for posterior characterization.

#### Components distribution

To evaluate the MSs matrix and protein distribution through MSs, DX-UDCA-GDNF-MSs formulation was elaborated incorporating a fluorescently labelled protein (fluorescein isothiocyanate-conjugated bovine serum albumin – BSA-FITC) as a model protein, and a fluorescent stain with affinity with the PLGA (Nile Red). To this, 3 µL of a Nile Red solution were added in the organic phase (4 mg/mL) and MSs elaboration was carried out as explained before substituting GDNF for BSA-FITC. These MSs were observed by confocal fluorescence microscopy (Leica TCS SP8, Wetzlar, Germany).

#### Encapsulation efficiency

For UDCA and DX quantification, 1 mg of MSs was dissolved in 2.5 mL of methylene chloride. UDCA and DX were extracted with 6 mL of ethanol or methanol, respectively, which also promotes polymer precipitation. Then, it was vortex mixed and centrifuged (5000 rpms, 5 min, 20 °C). The supernatant was filtered and analysed in LC/MS as described before. Each batch was quantified in duplicate.

In the case of formulation DX-UDCA-GDNF-MSs, the actives DX and UDCA were extracted as mentioned previously. On the other hand, to isolate GDNF, a liquid/liquid extraction was made as follows: 5 mg of MSs were dissolved in 0.7 mL of methylene chloride. Then, 0.7 mL of reagent diluent provided in the ELISA kit (1% BSA in PBS, pH = 7.4) were added and the system was vortex mixed. After that, the samples were centrifuged at 12,000 rpm, for 15 min at 4 °C and the aqueous phase was removed. According to previous studies [28], this procedure was carried out in a 4-total time, removing and replacing the aqueous phase to extract all the encapsulated protein. Afterwards GDNF concentration was determined by ELISA.

#### In vitro release studies

Triplicate samples of 5 mg DX-UDCA-MSs and DX-UDCA-GDNF-MSs were suspended in 2 mL of phosphate-buffered saline (PBS, pH 7.4, isotonized with NaCl) with sodium azide (0.02% w/v). The samples were kept at 37 °C under constant agitation in a water shaker bath (WNB Memmert Shaking Bath, Memmert, Schwabach, Germany). At pre-set times (24 h, 7 days, and once a week until the end of the study), the MSs were centrifuged (5000 rpm, 5 min, 20 °C). The supernatants were recovered, filtered (0.22 µm), and replaced with 2 mL of fresh PBS/azide. The UDCA and DX concentrations of the before-mentioned filtered supernatants were analysed by LC/MS as described previously. The same procedure was also carried out to evaluate the burst release, removing the supernatants and quantifying actives at 24 h.

Moreover, triplicate samples of 5 mg DX-UDCA-GDNF-MSs were suspended in 2 mL of the medium with the addition of BSA (1%w/v) according to previous studies [28]. The samples were kept at the same conditions and at the same pre-set times, the supernatants were extracted. GDNF concentrations were analysed by ELISA.

The similarity test (f_2_) was calculated to compare the different drug release profiles (Eq. 1). This equation is a logarithmic transformation of the sum-squared error of differences between the reference (R_t_) and the test (T_t_) formulations over all time points:

$${F}_{2}=50*log\left\{\frac{1}{\sqrt{1+\frac{\sum_{t=t}^{t=n}{\left({R}_{t}-{T}_{t}\right)}^{2}}{n}}}*100\right\}$$

Equation 1 – Similarity test (f_2_) to compare the dissolution profiles.

Where n is the number of experimental points, t is the time points, and R_t_ and T_t_ are the mean percentages of the dissolved drugs in the reference and test formulations, respectively. Time points with 85% or more of the dissolved drug was not considered. When an f_2_ value is in the range of 50–100 indicates that the two release profiles are similar [41].

Moreover, with the aim of analysing the possible mechanism involved in the drug release kinetics, the release experimental data were fitted in different kinetic models (See supplementary methods and tables) being fitted successfully in the *Gallagher-Corrigan model* [42] with the adjustments that Gorrasi et al. suggested [43]. Gallagher and Corrigan developed a mathematical model that describes sigmoidal shape profiles, that usually occur in drug delivery systems where polymer undergoes degradation. The profile is described by the following equation that comprises the initial burst release because of the non-bounded drug to the matrix, followed by a slow release due to matrix erosion.

Gorrasi et al. modified that equation by adding a constant parameter (b) that considers the initial burst release. This factor shifts the model predictions up to fit the experimental release data. In the case that burst release does not exist, the b value is zero, becoming the original release equation (Gallagher-Corrigan equation). This equation is described as follows [43]:

$$Y(t)=b+{Y}_{1}(1-{e}^{{-k}_{1}t})+{Y}_{2}\left(\frac{{e}^{{-k}_{2}({t}_{2}-t)}}{1+{e}^{{-k}_{2}({t}_{2}-t)}}\right)$$

In this case, Y(t) is the drug fraction released in a t time, Y_1_ and Y_2_ are the relative amounts of drug release in the first and second mechanisms, respectively; K_1_ and K_2_ are the kinetic constants of the first and second mechanisms, respectively; t_2_ is the characteristic time of the second step mechanism and b is the burst parameter. This equation possesses 7 unknown parameters that were determined by adjusting the equation to the experimental data releases, using MATLAB^®^ (MathWorks, USA).

In order to choose the model which fits best, the coefficient of determination is used (R^2^). However, when the models have different number of parameters, it is more meaningful to use the adjusted coefficient of determination (R^2^_adjusted_):

$$R_{\mathrm{adjusted}}^2=1-\frac{(n-1)}{(n-p)}(1-R^2)$$

where:

N: is the number of dissolution data points
P: is the number of parameters in the models
R^2^: is the coefficient of determination [44]

#### Microspheres degradation studies

5 mg of the final formulations (Blank-MSs, DX-UDCA-MSs, DX-MSs, UDCA-MSs, DX-UDCA-GDNF-MSs but substituting GDNF for BSA) were placed in a water bath with similar conditions as in vitro studies, to assess its degradation. Media was replaced every week by centrifuging 5000 rpms, 5 min and 20 °C, and at pre-set times (every 2 weeks) some samples were removed, washed thrice with MilliQ^®^ water, and freeze-dried. Then, samples were observed in SEM with the same conditions as before, and molecular weight was assessed by GPC. GPC was composed of an Agilent 1260 Infinity II LC system coupled to a light scattering detector (Polymer Laboratories, Church Stretton, UK). The columns used were 2 × Plgel Mixed-D (300 × 7.5 mm × 5 µm) (Polymer Laboratories, Church Stretton, UK) at 25ºC with a rate of 0.8 mL/min of THF. The injection volume was 20 µL. Samples were dissolved in 1 mL of THF and filtered through 0.45 µm.

#### Differential scanning calorimetry (DSC)

Thermal analysis of raw material (PLGA, DX, and UDCA), empty and drug-loaded MSs prepared with the different mixtures of organic solvents were made by a Mettler differential scanning calorimeter (DSC820, Mettler Toledo, Greifensee, Switzerland) connected to a TAC7/DX instrument controller. Between 5–10 mg samples were employed and an empty aluminium pan (Mettler Toledo, Greifensee, Switzerland) was used as a referenced standard. The samples were heated at a 10 °C/min rate in a heating–cooling-heating cycle where the temperature ranges were as follows 25–100 °C/100–25 °C/25–280 °C.

#### Gas chromatography (GC)

The remaining methylene chloride present in the loaded MSs prepared with the different organic solvent mixtures was measured by a Headspace method in Gas Chromatography Mass Spectrometry (GCMS). Microspheres samples (approximately 100 mg) were suspended in 10 mL of water. The samples were extracted by shaking at 250 rpms and 90 °C for 30 min. Then, 2 mL of the headspace were injected into the GC column. CP-SELEC 624 CD (30 m × 0.25 mm × 1.4 um) was used as a column and helium at 1 mL/min flow rate was employed as a carrier. The GC temperature was equilibrated to 33 °C for 6 min. Then, a gradient from 33 °C to 250 °C at 20 °C/min was used and the temperature was maintained for 3 min. In order to quantify the amount, a line with 5 different points was made.

#### X-Ray diffractometry analysis

MSs and the compound’s crystalline structure were assessed by an X-ray diffractometer (Philips X’Pert, USA). The angular range employed was from 5.0° to 50.0° (2θ) with a step size of 0.033° and a scan rate of 100.0 s/step.

### Experiments on animals

A longitudinal and interventionist study was used to evaluate the neuroprotective effect on neuroretinal degeneration (4 different cohorts of animals). A first cohort composed by healthy Long-Evans rats (n = 17), a second cohort by glaucomatous rats (n = 30) induced by a single intracameral injection of biodegradable PLGA-MSs loaded with fibronectin (for more information about FN-loaded MSs and glaucoma animal model readers are kindly suggested to read Munuera et al. (2023) [40]), a third cohort by glaucomatous rats (n = 20) (same hypertensive model used) also injected into vitreous (IV) with non-loaded PLGA-MSs, and a fourth cohort by glaucomatous rats (n = 20) (same glaucoma model used) and intravitreally treated with the PLGA-MSs multiloaded DX-UDCA-GDNF-MSs formulation selected from in vitro studies.

All work with animals was performed in accordance with the Association for the Research in Vision and Ophthalmology (ARVO) statement on the Use of Animals and was previously approved by the Ethics Committee for Animal Research (P179/20). The study was carried out in the experimental surgery department of the Biomedical Research Center of Aragon (CIBA), located in Zaragoza, Spain. A total of 87 Long-Evans rats (50% males/50% female) aged 4 weeks old and weighted ranging from 50–100 g at the beginning of the study were used. The environmental conditions were controlled: 12-h light/dark cycles, temperature of 22 ºC, relative humidity of 55%, and housed in standard cages with environment enrichment, water, and food ad libitum.

**Fig. 2:**
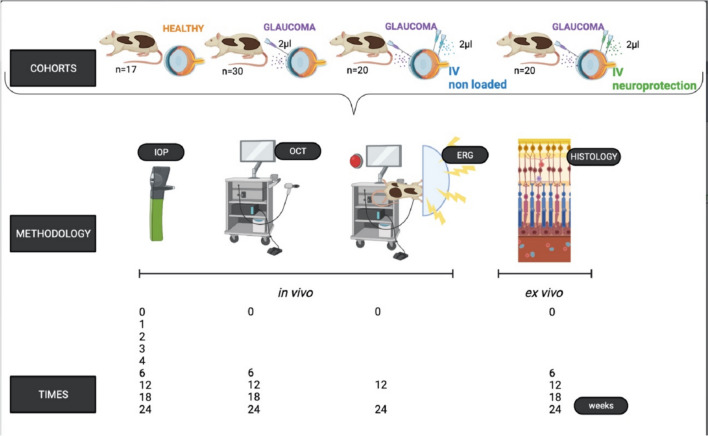
Work with animals. IV: intravitreal injection; IOP: intraocular pressure; OCT: optical coherence tomography; ERG: electroretinography. Created with BioRender.com

The single injections used for inducing the model of chronic glaucoma (2µL fibronectin-PLGA-MSs suspension) were all performed through the cornea into the anterior chamber of the right eye, using a micrometer Hamilton syringe with a glass micropipette at baseline. The injections for treatment (2µL DX-UDCA-GDNF-MSs suspension) were performed into the vitreous chamber at 2 and 12 weeks since the drugs delivery lasted up to 10 weeks in vitro. All injections were performed by specialists in Ophthalmology, under aseptic conditions. During procedures, temperature was controlled with warm pads, and after that, animals were left to recover in an oxygen-enriched (2.5%) atmosphere. Animals were followed throughout 24 weeks evaluating the effect on intraocular pressure (IOP) and neuroretinal functionality and structure by in vivo (electroretinography – ERG – and optical coherence tomography – OCT –) and ex vivo methods (histological studies). Figure 2. In vivo methodology on the animals was performed as in our previous works [40]. For details, please see Supplementary methods 2.

### Histology

After each OCT examination, the 6 animals were euthanized with an intracardiac injection of sodium thiopental (25 mg/mL) under general anesthesia, in accordance with humane conditions. Their eyes were immediately enucleated and fixed in paraformaldehyde 4%, 1 h at 4ºC. After fixation, eyes were progressively dehydrated by incubation in increasing alcohol concentrations, prior to embedding. Paraffin-embedded eyes were sectioned (5 μm) along the eye axis, deparaffinized, and rehydrated. Sections were incubated overnight at 4 °C with the following primary antibodies: anti-Brn3a (14A6, Santa Cruz Biotechnology), 1:50; anti-GFAP (Z0334, Agilent, Dako). Immunohistochemistry controls were performed by omission of the primary antibody in a sequential tissue section. After washes, slides were incubated with the required secondary antibodies followed by Hoescht (Thermo Fischer Scientific) for nuclei counterstaining. Slides were mounted in Shandon Immu-Mount (Thermo Fischer Scientific) medium for microscopic analysis. Microscopy was performed using the following systems: laser scanning confocal microscope TCS SP5 (Leica Microsystems), LSM 880 confocal microscope, and Axio Imager M2 (Carl Zeiss). Confocal image stacks were processed and quantified with the ImageJ.

### Statistical analysis

Data were recorded in an Excel database. Statistical analysis was performed using IBM SPSS version 22.0 (SPSS Inc, Chicago, IL).

The Kolmogorov–Smirnov test was used to assess sample distribution. Since the non-nonparametric distribution, a Mann–Whitney U test was used to evaluate differences between groups. Values were expressed as mean standard deviation. Values of p < 0.05 (marked with an *) were considered statistically significant. The Bonferroni correction for multiple comparisons was applied to avoid a high false-positive rate.

All data were analyzed by one-way analysis of variance (one-way ANOVA) using GraphPad Prism 8 (GraphPad Software Inc, San Diego, CA, USA). Results were presented as means ± standard deviation (SD) and P < 0.05 was considered significant.

## Results

### Optimization of the formulation

#### Morphological studies, particle size distribution, and drug distribution

**Fig. 3:**
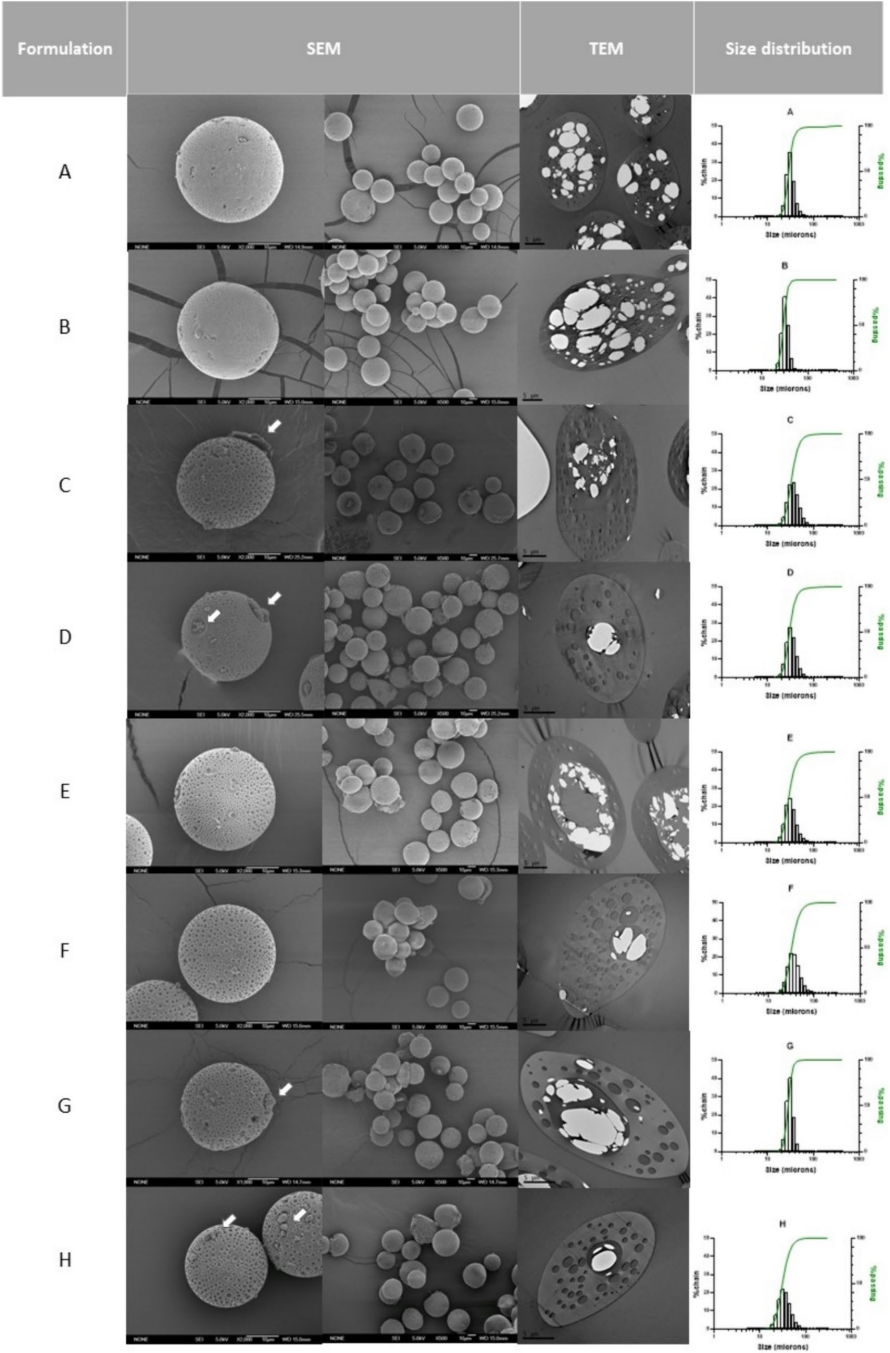
Scanning (SEM) and transmission (TEM) electron microscopies pictures and particle size distribution of the different MSs (**A-H**). Individual scanning pictures were made at × 2000, and group pictures at × 500. White arrows mark the presence of crystals

Figure 3 shows the internal and external morphologies of the different formulations. SEM images confirmed the spherical shapes and homogeneity in size. All formulations showed surficial pores and a slightly rough surface.

In the case of formulations A and B, prepared with methylene chloride, the presence of small pores covering the MSs surface was related to the addition of vitamin E in the formulation as previously described and it was not due to an inner porous structure [28, 30], as shown in TEM pictures (Fig. 3). On the contrary, according to SEM pictures, when microspheres were performed including the water-soluble organic solvent in the inner phase of the emulsion (EtOH), these surficial porous resulted clearly more numerous and bigger, and they were also observed through the polymeric matrix in the cross-sectional images from TEM.

Some of the preparations (C, D, G, and H) presented certain free drugs on their surfaces (white arrows in Fig. 3). This phenomenon was considered a key point to be avoided in the optimization procedure. TEM analysis reveals the presence of drug accumulations encapsulated into the microspheres for all formulations, however, the use of ethanol in the inner phase seemed to induce a different distribution of the drug in the polymeric matrix, so, for formulations C-H, drugs were mainly located in the core of the particles in contrast to the more generalized distribution that can be observed for formulations A and B, elaborated both using only methylene chloride as organic solvent of the inner phase.

The particle size distribution evaluation revealed that all formulations had a unimodal distribution in the selected range (20–38 µm).

#### Mean particle size

All the formulations had a mean particle size between 27.90 and 33.30 µm (Table 3). No significant differences among formulations were found regarding the solvent composition of the organic phase or the drug content.

**Table 3:** Mean particle size of the different developed formulations (A-H)

Formulation	Mean particle size (µm ± sd)
A	28.65 ± 0.70
B	29.11 ± 0.76
C	33.30 ± 2.03
D	29.84 ± 0.48
E	28.82 ± 1.02
F	32.27 ± 1.74
G	27.90 ± 0.76
H	30.02 ± 1.02

#### DSC studies

**Fig. 4:**
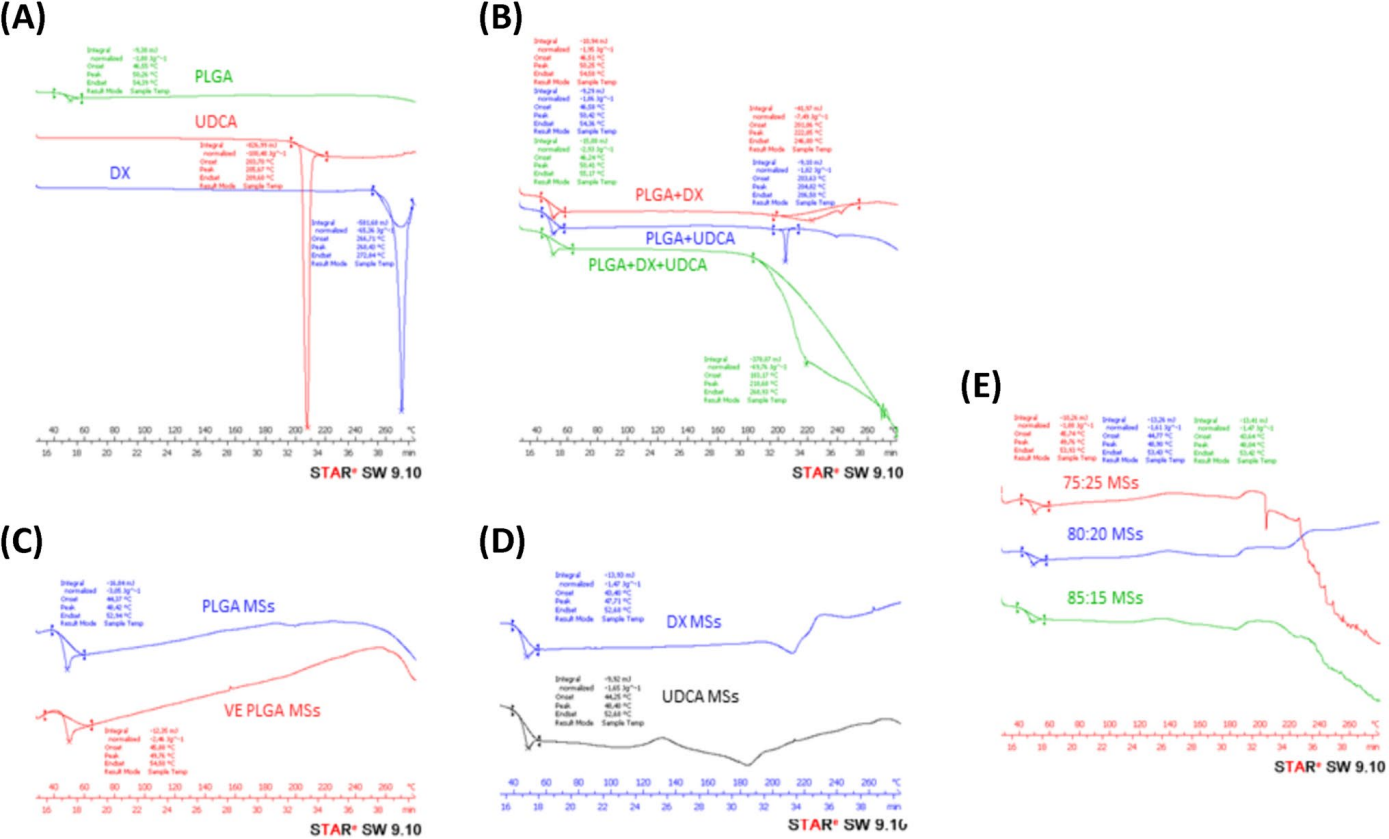
DSC curves of [**A**] PLGA (green), UDCA (red) and DX (blue), [**B**] physical mixtures of PLGA + DX (red), PLGA + UDCA (blue) and PLGA + DX + UDCA (green), [**C**] PLGA MSs (blue) and PLGA + vitamin E MSs (red), [**D**] DX MSs (blue) and UDCA MSs (black) and [**E**] DX-UDCA-MSs with different proportions of solvents 75:25 (red), 80:20 corresponding to **F** (blue) and 85:15 corresponding to H (green)

DSC curves of raw substances (PLGA, UDCA, and DX), different PLGA microspheres: PLGA, PLGA/vitamin E, and DX-UDCA-loaded PLGA/vitamin E microspheres prepared with different organic solvent proportions (75:25, 80:20, and 85:15) and physical mixtures containing the same drug-polymer fraction than microspheres are provided in Fig. 4.

When the raw substances were separately tested (Fig. 4[A]), the curve of PLGA showed a slight endothermic peak corresponding to its glass transition (50 °C), whereas the DX and UDCA curves showed sharp greater endothermic peaks corresponding to the melting points of their crystals (268 °C and 203 °C, respectively) as they are described in the literature [45, 46]. These peaks were also observed in the physical mixture (Fig. 4[B]).

Whereas in the case of formulations prepared with 80:20 MC:EtOH proportion, the melting endothermic peaks of drugs disappeared, suggesting a better drug dispersion into the polymeric matrix. In the case of formulations prepared with 75:25 and 85:15 MC:EtOH proportions, the profile decreased but it did not completely disappear (Fig. 4[D] and [E]), which might be attributed to the presence of drugs on the surface, as was expected in accordance with the images from scanning electron microscopy.

#### X-Ray diffractometry analysis

**Fig. 5:**
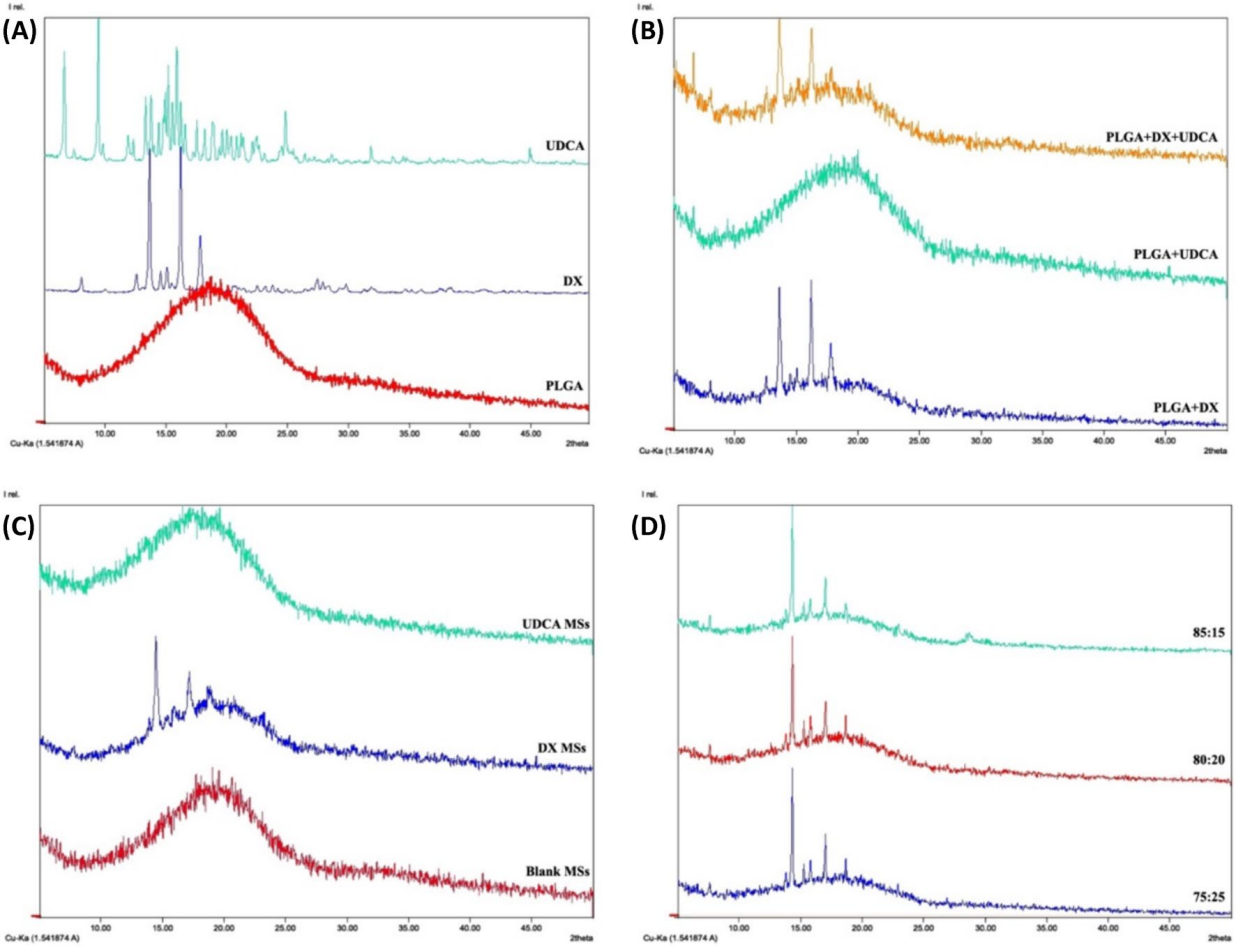
X-Ray diffractograms of [**A**] the different compounds (PLGA – red, DX – blue, and UDCA – green), [**B**] physical mixtures of the compounds (PLGA + DX – blue, PLGA + UDCA – green, and PLGA + DX + UDCA – orange), [**C**] non-loaded (red) and single loaded MSs (with DX – blue, with UDCA – green) and [**D**] co-loaded DX-UDCA-MSs elaborated with different solvent mixtures (MC:EtOH) (75:25 – blue, 80:20 – red and 85:15 – green)

The analysis by X-ray diffraction showed the presence of many diffraction bands in both drug’s powder samples reflecting their crystalline state. On the contrary, for the PLGA, no high intensity peaks were observed, corresponding to an amorphous state (Fig. 5[A]).

The characteristic peaks of DX in the physical mixtures and MSs samples suggest its incorporation in the MSs in the crystalline state. However, no UDCA characteristics peaks were observed, neither in the physical mixture nor in the MSs samples, probably because the PLGA signal could hide these peaks (Fig. 5 [B]). No differences in drugs spectra were observed in formulations prepared with different solvents proportions used in the organic phase of the emulsion with this X-ray diffraction analysis (Fig. 5[D]).

#### Gas chromatography

The headspace method in GCMS is a useful technique to quantify the content of organic solvent that remains after MSs manufacturing. Methylene chloride is a solvent of class 2, which means that its use must be limited. Both FDA and EMA limit its use to 600 ppm [47, 48]. However, ethanol is a 3-class solvent, meaning low toxic potential and its limits are wider. Thus, in this work, we only determined the residual content of methylene chloride in 100:0, 85:15, 80:20, and 75:25 MSs, finding in all of them a concentration of MC lower than 6 ppm/mg MSs (Table 4).

**Table 4:** Methylene chloride concentrations (ppm/mg MSs) of MSs elaborated with different dissolvent concentrations (100:0, 85:15, 80:20, 85:15 MC:EtOH)

MC:EtOH proportions	MC concentration (ppm/mg MSs)
100:0	3.61
85:15	3.89
80:20	4.56
75:25	5.34

#### Encapsulation efficiencies (EE)

Table 5 shows the values of entrapment efficiency of UDCA in the different formulations. To assess these data, the percentage of encapsulation has been statistically compared for those formulations that were made with the same initial amount of UDCA (40 mg). Thus, a statistically higher encapsulation has been observed for formulations C, E, F, and H, prepared including ethanol in different proportions in the internal phase of the emulsion, compared to formulation B (p < 0.01 for C, E and H and p < 0.001 for F), which was made exclusively using methylene chloride as organic solvent. No differences have been observed between the formulations prepared with different proportions of ethanol, so it seems that neither this parameter nor the co-encapsulation of different initial amounts of dexamethasone used on microspheres preparation had any influence on UDCA microencapsulation.

**Table 5:** UDCA entrapment efficiencies of the different formulations (A-H). A and B were elaborated only with methylene chloride in the inner phase of the emulsion while C-H were elaborated with different mixtures of methylene chloride and ethanol as solvent of the inner phase of the emulsion

Formulation	µg UDCA/mg MSs	Entrapment efficiency UDCA (%)
A	18.91 ± 2.95	45.08 ± 6.98
B	36.01 ± 0.87	45.73 ± 1.08
C	49.65 ± 2.78	63.08 ± 3.53
D	71.03 ± 4.11	63.00 ± 3.65
E	45.00 ± 3.09	61.38 ± 4.19
F	49.52 ± 7.53	65.15 ± 9.91
G	42.20 ± 1.46	57.57 ± 1.99
H	47.62 ± 2.03	62.50 ± 3.00

Table 6 summarizes the entrapment efficiency of DX in the different formulations. To treat these data, the formulations that were prepared with the same initial amount of dexamethasone have also been compared. In the case of those formulations initially prepared with 80 mg of dexamethasone, statistical differences were observed between formulations B and D (p < 0.01) and between formulations B and G (p < 0.05). However, it should not be forgotten that both formulations D and G presented drug crystals on their surface, according to SEM pictures and DSC thermograms, which could explain this increase in the quantified encapsulation percentage, which, therefore, does not have to represent the amount of drug that is included inside the microspheres.

**Table 6:** DX entrapment efficiencies of the different formulations (A-H). A and B were elaborated only with methylene chloride in the inner phase of the emulsion. C-H were elaborated with different mixtures of methylene chloride and ethanol as solvent of the inner phase of the emulsion

Formulation	µg DX/mg MSs	Entrapment efficiency DX (%)
A	123.64 ± 3.17	77.35 ± 2.09
B	117.25 ± 2.94	76.21 ± 1.94
C	140.68 ± 18.31	91.62 ± 11.93
D	148.93 ± 11.83	100.38 ± 8.54
E	131.45 ± 8.93	91.89 ± 6.28
F	95.79 ± 6.10	86.12 ± 5.49
G	133.95 ± 13.47	93.86 ± 9.57
H	92.15 ± 2.60	82.85 ± 2.34

On the other hand, the formulations prepared with 60 mg of dexamethasone (F and H), both elaborated including ethanol in the internal phase of the emulsion but in a different proportion, do not show significant differences in terms of the percentage of dexamethasone encapsulation, indicating that this parameter should not influence in the drug loading, despite of the fact that the drug is more soluble in EtOH:MC mixtures than in MC alone.

The objective of this first set of experiments was to establish better conditions to include all the drugs inside the microspheres. For that reason, the formulations that presented crystals on their surface according to SEM pictures and supported by DSC studies (C, D, G, and H) were discarded for further studies, regardless of the drugs encapsulation values obtained. Furthermore, A and B were also discarded for presenting the lowest values of % EE for both drugs, UDCA and DX.

#### In vitro release studies

A preliminary in vitro release study was performed with the formulations selected for further studies according to previous results (E and F). The release profile study of both drugs (UDCA and DX) was extended for 4 weeks. Since the burst release (understood as the amount of drug released in the first 24 h before becoming a stable release) is, together with the % EE, another crucial factor to optimize a drug delivery system, special attention was paid to this parameter (7).

**Table 7:** 24 h UDCA and DX burst release of selected formulations (E and F)

Formulation	µg UDCA/ mg MSs	UDCA released (%)	µg DX/ mg MSs	DX released (%)
E	17.64 ± 1.05	39.34 ± 3.28	26.60 ± 8.05	20.04 ± 4.87
F	8.78 ± 2.69	21.00 ± 4.48	18.13 ± 5.35	18.91 ± 5.50

**Fig. 6:**
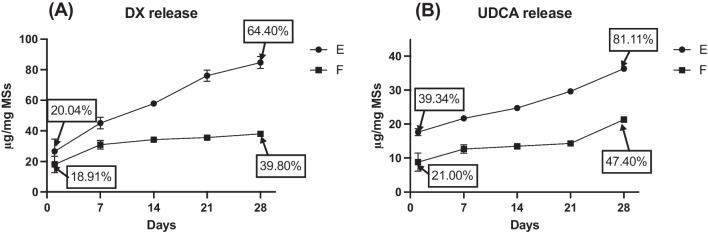
Cumulative in vitro release profiles (μg/mg MSs) of the formulations E and F for 28 days of DX (**A**) and UDCA (**B**). Formulation E was made with 80 mg DX and 80:20 MC:EtOH, F with 60 mg DX and 80:20 MC:EtOH

No statistical differences (p = 0.1639) were observed in the initial release (24 h) of dexamethasone from formulations E and F, even when different amounts of the anti-inflammatory drug were employed during microencapsulation. After that, as can be observed in Fig. 6A, a more rapid release of dexamethasone was observed for formulation E (2.15 µg DX/mg MSs/day) than for formulation F (0.74 µg DX/mg MSs/day). As for both formulations, the UDCA loading was statistically similar and they were prepared with the same MC:EtOH proportion, the differences found in dexamethasone release might be related to the higher loading of the drug observed for formulation E, in comparison with formulation F, according to Table 6 (131.45 ± 8.93 µg DX/mg MSs vs 95.79 ± 6.10 µg DX/mg MSs).

On the contrary, in the case of the more hydrophilic compound UDCA, statistical differences in the initial burst release were found (p < 0.05), although in both cases 40 mg was used for microencapsulation and the UDCA loading resulted similar. This fact could be due to the better distribution of UDCA into the PLGA matrix core in the presence of less initial amount of the corticosteroid. Interestingly, after this initial release, both formulations showed similar UDCA release rate values (0.69 µg UDCA/mg MSs/day for E and 0.46 µg UDCA/mg MSs/day for F).

The similarity test (f_2_) was evaluated for both drugs profiles, finding out DX and UDCA releases from both formulations were not similar (25.80 and 45.56 values, respectively), however in the case of UDCA release these differences were observed only in the initial release.

According to the data obtained until this point, and keeping in mind that the goal of this optimization process is to obtain a microparticulate delivery system able to co-release the active compounds for several months, formulation F, from now named as “DX-UDCA-MSs” was able to promote a slower release of both UDCA and DX and was the one selected as platform for further protein microencapsulation.

### Protein inclusion in the optimized formulation

#### Morphological studies, mean particle size, and particle size distribution

**Fig. 7:**
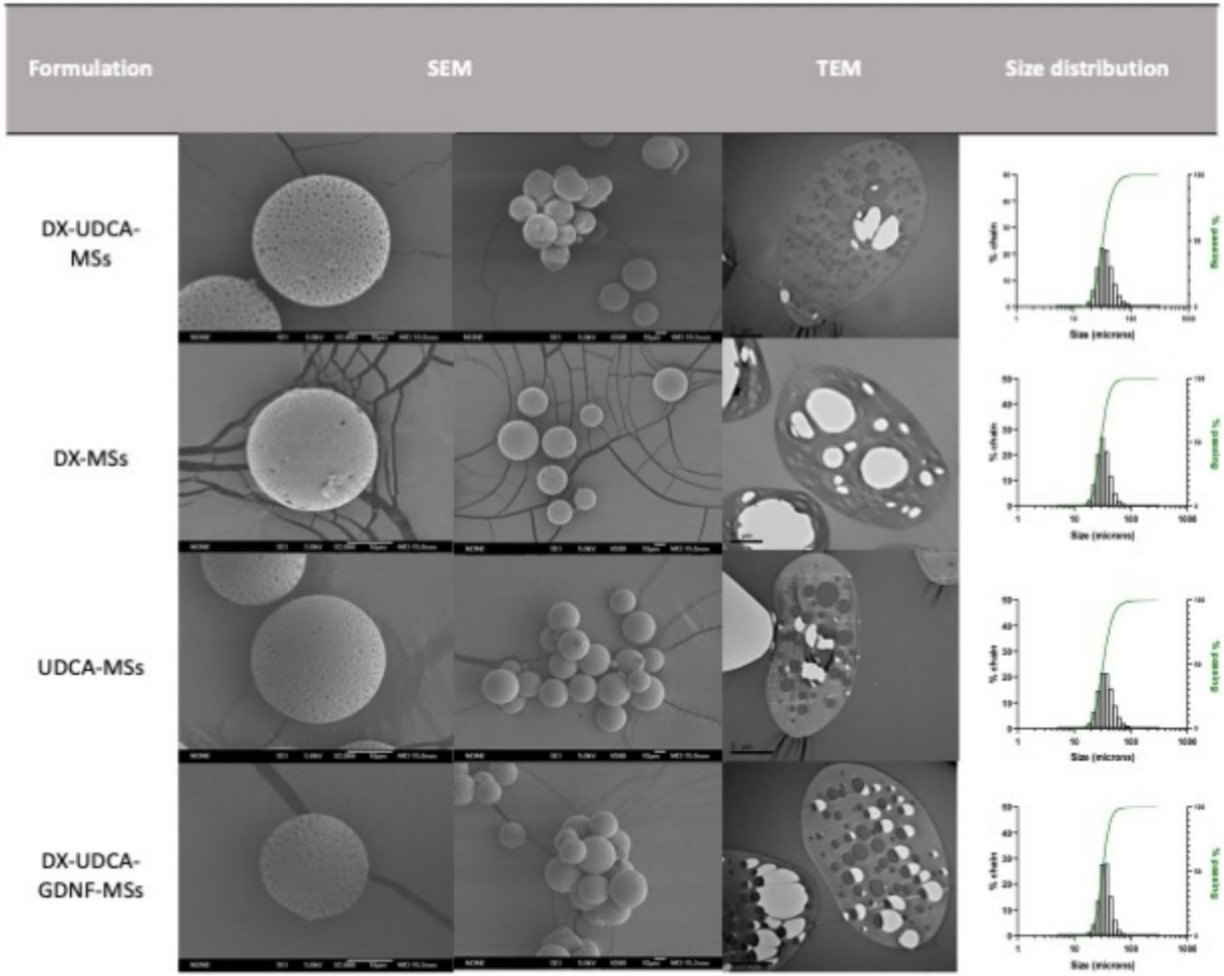
Scanning and transmission electron microscopies pictures and particle size distribution of the selected formulation (DX-UDCA-MSs), the only loaded DX formulation (DX-MSs), the only loaded UDCA formulation (UDCA-MSs), and the selected formulation with protein included (DX-UDCA-GDNF-MSs) MSs. Individual scanning pictures were made at × 2000, and group pictures at × 500

As can be seen in Fig. 7, all the formulations presented homogeny with spherical shapes, the presence of surficial pores, and a slightly rough surface, in accordance with the previous results. The pores can be observed through the polymeric matrix thanks to the TEM images. These pictures also confirmed the presence of drug accumulations in all the MSs, not only in the multi-loaded formulations but also in the MSs loaded with only one active compound (DX-MSs and UDCA-MSs).

All formulations had an unimodal distribution of the particle size in the selected range (20–38 µm) (Fig. 7) with similar mean particle size in all cases (between 29.49 and 32.29 µm) (Table 8).

**Table 8:** Mean particle size of the selected DX-UDCA-MSs formulation (F), the formulations with only DX or UDCA included (DX-MSs and UDCA-MSs) and the final formulation with GDNF included (DX-UDCA-GDNF-MSs)

Formulation	Mean particle size (µm ± sd)
DX-UDCA-MSs	32.27 ± 1.74
DX-MSs	29.49 ± 0.39
UDCA-MSs	32.29 ± 0.51
DX-UDCA-GDNF-MSs	31.76 ± 0.83

#### Matrix structure and protein distribution

**Fig. 8:**
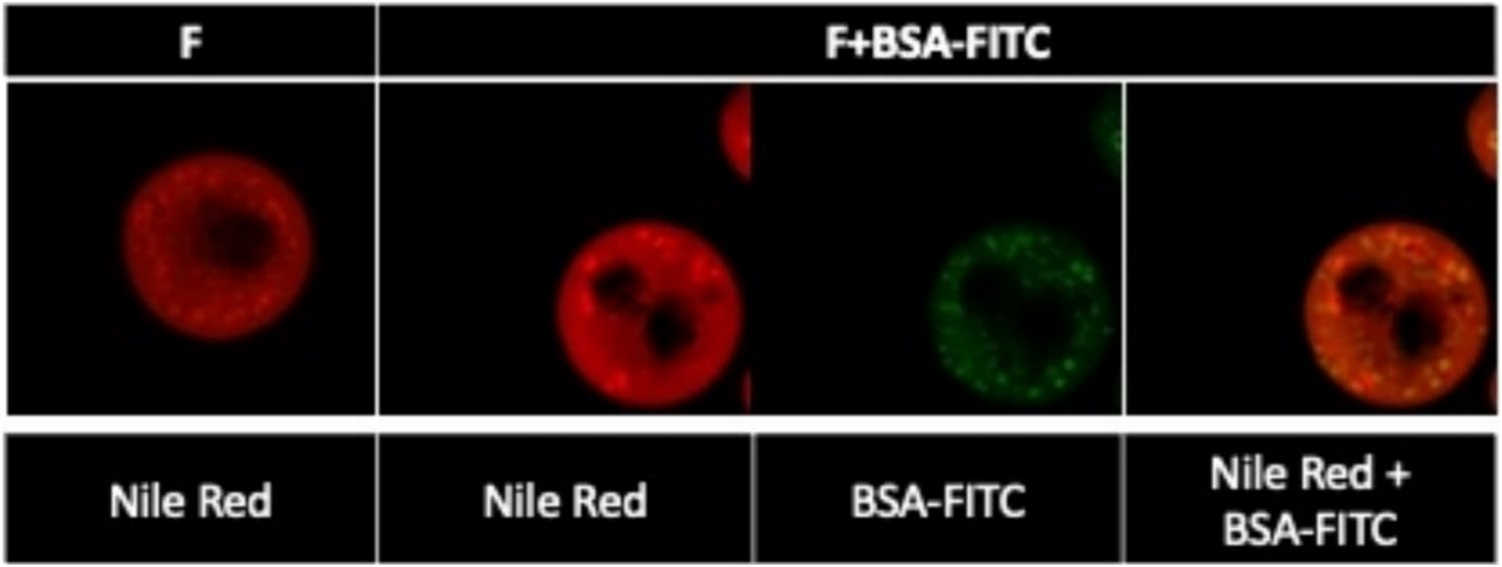
Confocal microscopy images of Nile Red labelled PLGA microsphere and BSA-FITC distribution. From left to right: microspheres of formulation DX-UDCA-MSs, microspheres of formulation DX-UDCA-GDNF-MSs replacing GDNF for BSA-FITC (Nile Red channel, BSA-FITC channel, and the combination of Nile Red and BSA-FITC channels)

To study the distribution of the protein, batches with the same composition of formulations DX-UDCA-MSs (F) and DX-UDCA-GDNF-MSs were elaborated including Nile Red in the polymeric matrix and replacing GDNF for BSA-FITC in formulation DX-UDCA-GDNF-MSs. They were observed by confocal microscopy (Fig. 8). It is well known that Nile Red possesses a high affinity for the PLGA, so the absence of red could be due to the presence of pores, but also to active compounds accumulations in solid-state. On the other hand, BSA-FITC is distributed heterogeneously through the microsphere, being a higher fluorescence located near the surface.

#### Encapsulation efficiencies

Table 9 compiles the encapsulation efficiencies of the different substances in the initially optimized, mono-loaded, and protein-loaded MSs. No significant differences were found between mono-loaded and double or tri-loaded MSs in terms of drug loading. It is worth mentioning that the entrapment efficiency of each one of the three substances in the tri-loaded formulation (DX-UDCA-GDNF-MSs) was above 50% of the theoretical amount.

**Table 9:** Entrapment efficiencies of the different active compounds (DX, UDCA, GDNF) in the mono-loaded, optimized, and final formulations

Formulation	Entrapment efficiency UDCA (%)	Entrapment efficiency DX (%)	Entrapment efficiency GDNF (%)	Entrapment efficiency UDCA (µg/mg MSs)	Entrapment efficiency DX (µg/mg MSs)	Entrapment efficiency GDNF (ng/mg MSs)
DX-UDCA-MSs	65.15 ± 9.91	86.12 ± 5.49	-	49.52 ± 7.53	95.79 ± 6.10	-
DX-MSs	-	79.36 ± 1.37	-	-	103.74 ± 1.83	-
UDCA-MSs	59.79 ± 5.38	-	-	55.55 ± 4.98	-	-
DX-UDCA-GDNF-MSs	64.27 ± 1.34	84.36 ± 1.80	51.60 ± 1.10	52.65 ± 1.12	101.04 ± 2.17	19.83 ± 0.42

#### In vitro release studies

**Fig. 9:**
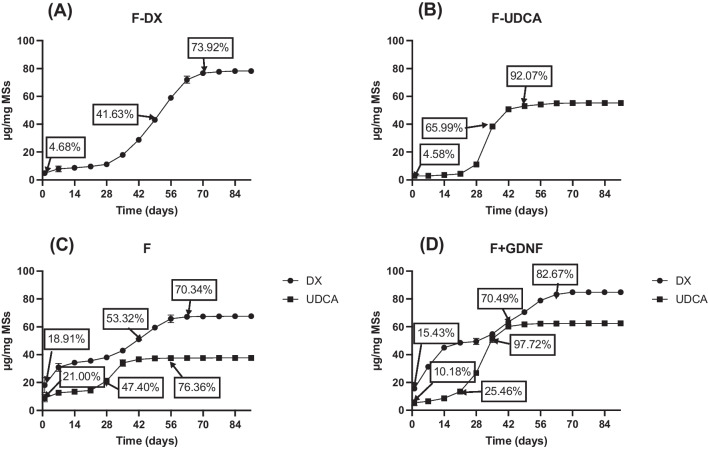
Cumulative in vitro release (μg/mg MSs and %) of DX from DX-MSs (**A**), of UDCA from UDCA-MSs (**B**), of DX and UDCA from DX-UDCA-MSs (**C**), and of DX and UDCA from DX-UDCA-GDNF-MSs (**D**)

A prolonged in vitro study was carried out with the mono-loaded (DX-MSs and UDCA-MSs), the double (DX-UDCA-MSs), and the tri-loaded MSs (DX-UDCA-GDNF-MSs). According to Fig. 9, multiphasic profiles for both substances (DX and UDCA), characteristics of PLGA microspheres, where slow and fast release rates were alternating, were observed until the end of the release assay (91 days) (Fig. 9).

**Fig. 10:**
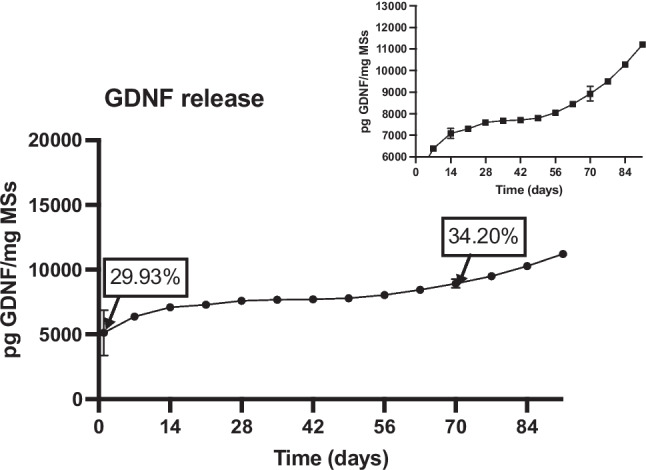
Release profile of GDNF from DX-UDCA-GDNF-MSs. A more detailed zoom of the release has been inserted from day 7 until the end of the study (91 days)

As expected after confocal microscopy observations, the GDNF delivery study (Fig. 10) showed an important initial release (24 h burst effect) (5125.81 ± 1745.71 pg GDNF/mg MSs), due to the presence of the protein near the MSs surface. After that, a multiphase release rate profile was also observed during the 91 days of the in vitro release study.

As described in the supplementary materials and methods section, different equations were applied to the experimental release data in order to fit them in a kinetic model in an attempt to explain the influence of the co-loading and co-release on the release mechanism. Supplementary tables 1, 2, and 3 summarized the statistical data for a more accurate analysis.

For zero- and first-order, Korsmeyer-Peppas, Higuchi, Baker-Lonsdale, and Weibull models, it was not possible to fit the experimental data in only one time-step. On the contrary, it was necessary to split the release profile into different steps (2 or 3) and even in that case, none of them showed an accurate adjustment for these models (R^2 ^< 0.97) (see complementary data). The Gallagher-Corrigan models (with or without Gorrasi correction) showed the best adjustment for all the release profiles (DX, UDCA, and GDNF) with R^2^_adjusted_ above 0.989 for the whole profile. This model describes the sigmoidal shape of the release profile by two stages: (stage 1) what authors call a “burst effect” step, but that in this model does not only consider the 24 h initial release as usual, but a more complex and prolonged process in which the “non-bonded” drug is released, and (stage 2) a “matrix erosion” step, in which the remaining drug in the system is released as the polymer degrades and therefore the matrix is eroded. In addition, when the data is fitted using the Gorrasi adjustment, very similar results are observed between the "calculated B" values and the "experimental B" values, the latter obtained as the fraction of drug released in the first 24 h in the in vitro release studies. Table 10 shows the different parameters values in the final model.

**Table 10:** Values of the release model parameters in the different in vitro experiments. (Experimental B – 24-h release of experimental data –, B – burst parameter from the equation –, Y_1_ and Y_2_ – relative amounts of drug release in the different stages –, K_1_ and K_2_ – kinetic constants of the different stages –, and t_2_ – characteristic time of the stage 2 where the maximum drug is released –)

Samples	Experimental B	Stage 1			Stage 2		
		B	Y_1_(-)	K_1_(day^−1^)	Y_2_	K_1_(day^−1^)	T_2_(day)
*UDCA release*							
UDCA-MSs	0.0458	0.0408	0.2000	0.0087	0.8550	0.3760	32.80
DX-UDCA-MSs	0.2100	0.1442	0.1264	0.3027	0.4866	0.3498	30.03
DX-UDCA-UDCA-GDNF-MSs	0.1018	0.0882	0.1568	0.0469	0.9481	0.2997	30.37
*DX release*							
DX-MSs	0.0468	0.02982	0.0468	0.4090	0.6986	0.1337	49.15
DX-UDCA-MSs	0.1891	0.1436	0.2225	0.2284	0.3442	0.1661	42.25
DX-UDCA-GDNF-MSs	0.1543	0.1066	0.3954	0.1157	0.3442	0.1537	46.71
*GDNF release*							
DX-UDCA-GDNF-MSs	0.2993	0.2648	0.1524	0.1185	0.2688	0.0885	81.07

According to this model, there is no difference in the fraction of UDCA released controlled by dissolution processes (stage 1), (the addition of B and Y_1_ parameters), regardless of the presence of dexamethasone (UDCA-MSs vs DX-UDCA-MSs and vs DX-UDCA-GDNF-MSs), although it seems that the co-microencapsulation of this hydrophobic drug (DX-UDCA-MSs and DX-UDCA-GDNF-MSs formulations) induces a greater presence of UDCA in the vicinity of the surface of the microspheres, and therefore a greater release in the first 24 h (B: 0.041 for F-UDCA, 0.144 for F – the co-loaded formulation – and 0.089 for F + GDNF – the tri-loaded formulation). Furthermore, the release rate during stage 1, as mentioned mainly due to dissolution processes and given by the kinetic parameter K_1_, showed a lower rate of UDCA release when there is neither dexamethasone (K_1_: 0.303 day^−1^ for DX-UDCA-MSs vs K_1_: 0.008 day^−1^ for UDCA-MSs) nor GDNF (K_1_: 0.047 day^−1^ for DX-UDCA-GDNF-MSs vs K_1_: 0.008 day^−1^ for UDCA-MSs) in the formulation. Once the second stage of release is reached, controlled by mechanisms related to the degradation of the polymer according to this model, there seems to be no influence of co-microencapsulation, with a similar higher release rate in all three cases.

On the other hand, when dexamethasone release profiles are studied using this kinetic model, the fraction of drug released in stage 1, governed by the dexamethasone dissolution rate according to the model, is clearly increased by the co-microencapsulation of UDCA (DX-UDCA-MSs and DX-UDCA-GDNF-MSs formulations), considering both the 24-h release (B) and the relative amount delivered in this phase (Y_1_). As observed for UDCA release data, in the second stage of release, governed by polymer degradation mechanisms according to Gallagher and Corrigan [42], the influence of co-microencapsulation is less marked.

Finally, regarding the kinetic data for GDNF, an important release is described at 24 h (B), which is consistent with the arrangement of the protein in the vicinity of the surface of the microspheres observed in confocal studies, followed by a slow release of the remaining protein, both in the stage governed by dissolution processes and in the stage controlled by polymer degradation processes.

#### Degradation

**Fig. 11:**
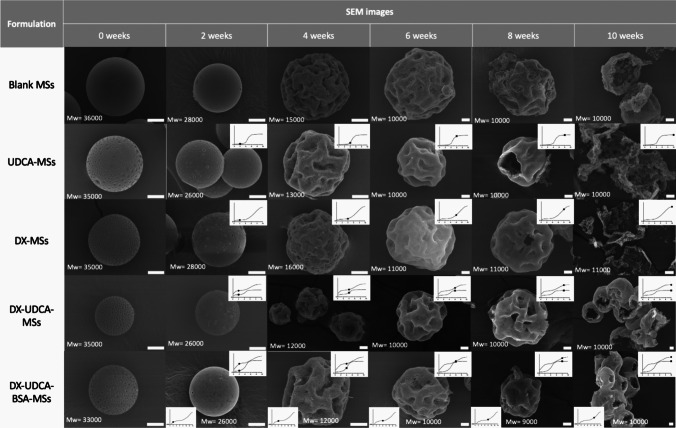
SEM images from different degradation dates (0, 2, 4, 6, 8, and 10 weeks) of different formulations (Blank MSs, UDCA-MSs, DX-MSs, DX-UDCA-MSs and DX-UDCA-BSA-MSs) with the PLGA molecular weight. Scale bars: 10 µm

In order to complete the release study, degradation studies were carried out. As can be seen in Fig. 11, all batches at time 0 presented a “golf-like” surface due to the presence of vitamin E [28] and it became smoother at week 2. It seemed to be very crucial, for the release of DX and UDCA, the period between 4 and 6 weeks, when microspheres lost their spherical structure – having a spheroid morphology – and started to appear more cavities. According to in vitro release profiles, during this period, the release of DX and UDCA increased (that corresponds to the T_2_ value), being sooner and more acute in the case of the UDCA. In this phase, the degradation stage in the kinetic model started. Moreover, it seems that the formulation exclusively made with dexamethasone presents shallower cavities after 4 weeks than the rest of the formulations in which there is UDCA, which would be in accordance with the hypothesis proposed after the kinetic treatment that the co-microencapsulation with UDCA enhances the release of dexamethasone in stage 1.

At week 8, all batches showed big pores, corresponding with the fast release of the protein. However, for the other compounds, the release had finished. Finally, at week 10, the microspheres structure collapsed.

### Experiments on animals

#### Intraocular pressure

**Fig. 12:**
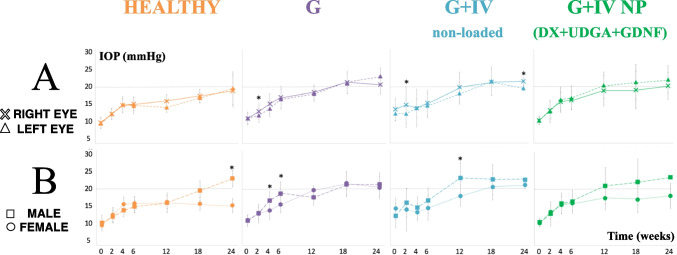
Intraocular pressure (IOP). Comparison of intraocular pressure curves in the four cohorts. Subanalysis by eye laterality (**A**) and by sex (**B**) in each model. *: statistical significance (p < 0.05). G: Glaucoma cohort; IV: intravitreal injection; NP: neuroprotective; DX: dexamethasone; UDCA: ursodeoxycholic acid; GDNF: glial cell line-derived neurotrophic factor

The right injected eyes of the G and G + IV unloaded cohorts had significantly higher IOPs over time. However, the G + IV neuroprotective multiloaded (DX-UDCA-GDNF) cohort showed a trend of lower values (Fig. 12A). A subanalysis by sex revealed different IOP behavior in each cohort. Males had higher IOP values than females, in the G cohort at 4 and 6 weeks, in the G + IV-unloaded cohort at 12 weeks, and in the healthy cohort at 24 weeks. However, in the treated cohort (G + IV neuroprotective multi-loaded—DX-UDCA-GDNF -) no statistically significant sex differences were found (Fig. 12B).

#### Electroretinographic analysis

The right eyes induced with glaucoma (G, G + IV unloaded, G + IV (DX-UDCA-GDNF) cohorts) showed worse signal compared to left eyes and the healthy cohort. The G cohort showed lower bipolar cell amplitude and RGC with progressive decreasing trend from 12 to 24 weeks. The G + IV unloaded cohort showed lower photoreceptor amplitude at 12 weeks, and bipolar cells throughout the study (at 12 weeks in ERG and at 24 weeks in PhNR). The G + IV neuroprotective multiloaded (DX-UDCA-GDNF) cohort showed lower photoreceptor amplitude throughout the study, and lower amplitude with higher bipolar cells latency at 24 weeks (in ERG and PhNR). However, RGC functionality increased from 12 to 24 weeks (PhNR wave: 16.60 ± 21.72 vs 20.97 ± 10.13 μV). Impaired and untreated cohorts (G and G + IV unloaded) had the longest latencies, and healthy and treated cohorts (G + IV neuroprotective multiloaded—DX-UDCA-GDNF -) the shortest latencies. The G + IV neuroprotective multiloaded (DX-UDCA-GDNF) cohort showed the highest RGC functionality of all at the end of the study (PhNR wave: Healthy: 11. 75 ± 11.94 μV vs G: 17.83 ± 17.66 μV vs G + IV unloaded: 18.33 ± 14.84 μV vs G + IV neuroprotective multiloaded—DX-UDCA-GDNF -: 20.97 ± 10.13 μV). A subanalysis by sex showed that males in the G cohort had lower bipolar amplitudes throughout the study, lower photoreceptor amplitudes at 24 weeks, and tendency to lower RGC functionality with decrease over time. However, in the G + IV unloaded and G + IV neuroprotective multiloaded (DX-UDCA-GDNF) cohorts, no sex differences were found (Supplementary Table).

#### Optical coherence tomography

**Fig. 13:**
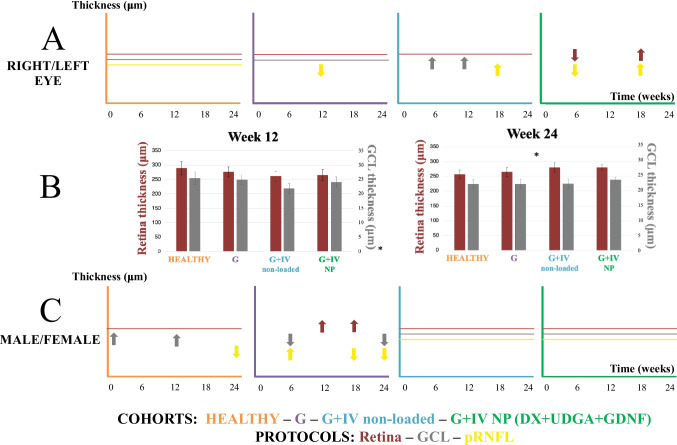
Optical coherence tomography (OCT). Comparison of retina, GCL, and pRNFL thickness in the four cohorts. **A**) Subanalysis by eye laterality in each cohort. **B**) Right eyes in retina and GCL protocols statistically significant in the four cohorts. **C**) Subanalysis by sex in each cohort. G: Glaucoma cohort; IV: intravitreal injection; NP: neuroprotective; DX: dexamethasone; UDCA: ursodeoxycholic acid; GDNF: glial cell line-derived neurotrophic factor. Horizontal lines mean no differences between the parameters analyzed. Up arrows mean an increase in thickness of right eye vs left eye and male vs female. Down arrows mean a decrease in thickness of right eye vs left eye and male vs female

The right eyes of cohort G showed lower pRNFL thickness (week 12). However, the G + IV non loaded cohort showed greater thickness in GCL (week 6 and 12) and pRNFL (week 18). In contrast, the G + IV neuroprotective multiloaded (DX-UDCA-GDNF) cohort presented lower thickness in retina and pRNFL at week 6, but higher thickness at week 18, and trend to lower in GCL at end times (Fig. 13 A). When comparing the four cohorts with each other, the ones with damage (G, G + IV unloaded and G + IV neuroprotective multiloaded—DX-UDCA-GDNF -) and especially with intravitreal injection, presented lower retinal and GCL thicknesses at week 12, but greater at week 24, with respect to the healthy cohort (Fig. 13B). Subanalysis by sex showed in the G cohort evident and multiple differences at all study time points. In general, males had higher retinal thicknesses (12 and 18 weeks) but lower GCL thicknesses (6 and 24 weeks), and higher pRNFL thicknesses at early time points (6 weeks) but lower at late time points (18 and 24 weeks). The G + IV unloaded and neuroprotective multiloaded (DX-UDCA-GDNF) cohorts showed no statistically significant differences by sex (Fig. 13C).

#### Histological analysis of the neuroretina

**Fig. 14:**
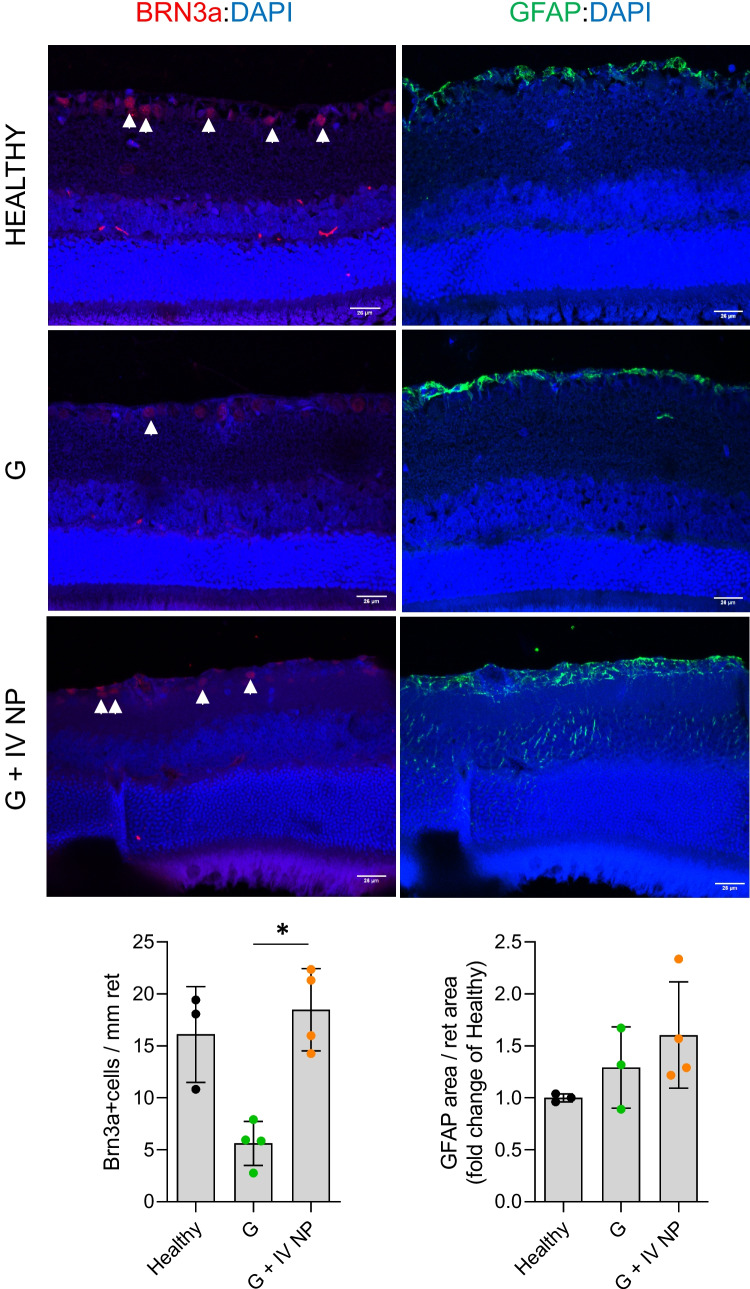
Histological analysis of retinas 24 weeks post-injection. Comparison of RGC count (Brn3a staining, left column) and astrogliosis (GFAP staining, right column) in the three cohorts. Healthy: cohort non-injected, G: glaucoma, G + IV NP: glaucoma + neuroprotection. *: statistical significance (p < 0.05)

To confirm the neuroprotective effect of the treatment, RGC number was determined in retinal histological sections from the Healthy, Glaucoma, and Glaucoma with neuroprotective treatment cohorts 24 weeks after the induction of glaucoma. Indeed, a significant increase in RGC counts was observed in the treated cohort when compared with the untreated glaucomatous cohort (Fig. 14, left column). Interestingly, the number of RGC in these animals was similar to that of age-matched healthy rats. Astrogliosis, evaluated as the expression of GFAP, remained unchanged amongst groups (Fig. 14, right column), with a tendency to be higher in the treated cohort, probably due to multiple injections.

## Discussion

Despite the advantages that the creation of IODDS loaded with more than one neuroprotective agent would entail from the point of view of the quality of life of patients and the burden on the health system [49], this technological strategy is very poorly developed, probably due to the complexity of including substances of different nature in the same formulation and ensuring control of their release. Indeed, the co-encapsulation of active compounds of different natures in the same microsystem is a major technological challenge. As mentioned, it is necessary to fine-tune the microencapsulation process to optimize not only the load of each of the compounds but also their subsequent controlled release. In this work, the starting point was the use of dichloromethane, widely employed in the preparation of PLGA microspheres as the only organic solvent for the internal phase of the emulsion in the solvent extraction/evaporation method [50]. However, it was also preferred to test the inclusion of ethanol as a co-solvent for the drugs to enhance their solubilization in the internal phase [51] and, at the same time, to promote faster precipitation of the polymer that would also improve their entrapment [52]. Indeed, this modification managed to increase the percentage of encapsulation of both UDCA and DX. However, the physicochemical characterization of the different batches by electron microscopy and DSC revealed the presence of drug crystals, probably DX according to RXD studies, on the surface of the microspheres that were prepared using a combination of MC:EtOH of 75:25 and 85:15 as solvents in the inner phase of the emulsion. This phenomenon is widely reported in the scientific literature and can be explained by the rapid diffusion of the drug to the surface of the PLGA microsphere when ethanol is included as a co-solvent in the MC solution up to a percentage [53]. The fact that these surface crystals were observed when 25% or 15% of ethanol was included in the organic phase but not when 20% of the co-solvent was used, might suggest that not only the ratio MC:EtOH but also, the co-microencapsulation of several active compounds in the same system might play an important role. For the system proposed, the formulations employing a ratio of 80:20 (MC:EtOH) seemed to be the balanced proportion between fast and slow solidification and drugs solubility [51, 52], not presenting crystals on their surfaces.

The “golf-like” MSs surface observed in all formulations was previously explained by the presence of the oily nature of vitamin E, which might induce a slow and homogeneous diffusion of the remaining solvent from the droplet in the latest steps of the maturation process [28, 30]. However, for formulations prepared including EtOH as co-solvent, bigger and more numerous porous were observed on the MSs surface, probably due to its rapid extraction and subsequent polymer matrix hardening of external layers of the droplet in contact with the aqueous media. This rapid hardening might also explain the appearance of pores through the polymeric matrix [52] and the distribution of drugs mainly in the core of the microspheres, observed in TEM pictures, and suggested from preliminary in vitro release studies.

Once the DX and UDCA co-microencapsulation procedure was optimized, the following step was the inclusion of the active protein in its solid state dispersed in vitamin E. For comparison reasons also formulations loaded only with dexamethasone and only with UDCA were prepared. Confocal studies demonstrated that the protein was mainly located close to the vicinity of the particles, which was already observed in previous studies and attributed to its displacement promoted by solvent extraction [28, 32]. Concerning the drugs encapsulation efficiencies, the final formulation achieved encapsulations over 50% of the initially incorporated amount for each component. Moreover, when the protein was combined, the encapsulation efficiencies of the other components did not change. To our awareness, this is the first tri-delivery formulation incorporating two low molecular weight compounds and a protein. It is worth mentioning that all MSs formulations evaluated showed residual solvent contents far below the allowed limits.

The release of DX and UDCA followed the typical behaviour of PLGA microspheres with different fast and slow releases, independently of the incorporation of one or more drugs. The formulation selected was able to release in a sustained manner DX until day 70, UDCA until day 70, and GDNF until day 91, which makes the proposed microspheres very suitable as intraocular drug delivery systems for neuroprotective purposes.

It can be found in scientific literature a wide number of equations and models useful to describe the release behaviour from drug delivery systems. Some models explain the drug release by simple matrix diffusion mechanisms such as Higuchi's model [44]. Others describe the release profile only by polymer degradation [54]. Based on these, other models have been developed to explain the release profiles due to the evolution of the system through time, combining these mechanisms with the burst effect [42]. Depending on the system under study, it is not always easy to find kinetic models capable of describing the different processes involved in the drug release from controlled release systems over long periods, since the mechanisms involved can change throughout the process. This kinetic evaluation is even more complicated when there is more than one active ingredient in the formulation. In fact, there is hardly any scientific literature in which this topic is addressed. In our study, for zero- and first-order, Korsmeyer-Peppas, Higuchi, Baker-Lonsdale, and Weibull models it was possible to fit the experimental only after splitting the release profile into 2 or 3 different steps. This could be easily explained by the fact that the release mechanism from sustained drug delivery systems can change during the release process. On the contrary, the Gallagher-Corrigan models (with or without Gorrasi correction) showed the best adjustment for the whole profiles (DX, UDCA, and GDNF) with R^2^_adjusted_ above 0.989. According to this model, in all cases two stages in the release profile can be detected, the first one governed by the drug dissolution and the second one by the polymer degradation. According to the data obtained in this work, it seems that the presence of both actives in the formulation mainly influenced the first stage. It can be hypothesized that the presence of dexamethasone does not influence the proportion of UDCA released governed by dissolution (stage 1) although it does influence the distribution of UDCA in the microsphere, increasing the release at 24 h. On the other hand, the presence of UDCA did increase the proportion of dexamethasone released by processes governed by dissolution and, also, the drug release rate, probably due to its amphiphilic nature or to the formation of bigger cavities in the PLGA matrix.

To evaluate the efficacy of the multi-loaded formulation, 4 cohorts of animals were compared (healthy vs glaucoma vs glaucoma + IV unloaded vs glaucoma + IV multi-loaded formulation) and the influence of sex and bilaterality was analyzed over 6 months of study. The amount of microspheres to be administered in the posterior segment of the eye of the rats was 0.1 mg (intravitreal concentration of 2 mg/mL), established as safe in previous studies by the research group [55].

Intravitreal injection did not produce a significant increase in IOP, relative to the injection volume per se. However, at the end of the study (after two intravitreal injections), a clinically significant late increase in IOP was detected as the G + IV unloaded cohort showed a trend of lower neuroretinal thicknesses and even worse functionality than G cohort. Interestingly, the cohort treated with the IV neuroprotective multiloaded formulation showed a trend of lower IOP values. These data suggest that the formulation might have some IOP control effect, despite not being composed of any drug considered to be hypotensive. The lower RGC death observed in the glaucomatous cohort treated with the neuroprotective formulation could also decrease the overall inflammation of the eye [56–62] and prevent IOP increase. In all cohorts in our study, males tended to have higher IOP values. This is consistent with the higher IOP levels found in male rats [63] and human males [64]. However, in the cohort treated with the neuroprotective formulation, no significant sex differences were found, suggesting that the proposed neuroprotective multiloaded formulation could be useful in both sexes.

To evaluate the efficacy of the proposed formulation, neuroretina was analyzed in vivo by noninvasive technology by ERG and OCT, and ex vivo by histology.

Eyes with glaucoma showed worse functionality, even with bilateralization and coincident with increasing IOP. The G + IV unloaded cohort showed even worse functionality, suggesting further damage resulting from repeated injections (anterior chamber and intravitreal) [65, 66].

However, the G + IV neuroprotective multiloaded (DX-UDCA-GDNF) cohort even with the same repeated injections improved RGC functionality as measured by the PhNR test at end times. Cohorts with damage (especially males) presented worse functionality than healthy and neuroprotective formulation-treated cohorts, reflecting the maintenance of RGC functionality that coincides with the higher and similar number of RGCs counted in histological studies.

In relation to structural neurodegeneration, OCT evaluation showed lower thicknesses in the glaucoma-induced eye at early and intermediate times. The G and G + IV unloaded cohorts showed the lowest thicknesses in pRNFL, reflecting induced and untreated damage, at week 24. Interestingly, G + IV neuroprotective multiloaded (DX-UDCA-GDNF) cohort showed a trend toward greater retinal and pRNFL thicknesses at end times. This increased thickness measured by OCT could be related to the neuroprotection detected with higher RGC counts, but also, in part, to the increased gliosis observed in histological studies at late times. The G + IV neuroprotective multiloaded (DX-UDCA-GDNF) cohort showed similar GCL and pRNFL thicknesses to the healthy cohort at 12 weeks, and the greatest thicknesses at 24 weeks. Furthermore, the neuroprotective effect seems to be maintained more in females, as they showed protection in GCL and pRNFL up to 24 weeks; a milestone that in males occurred at 12 weeks but was only maintained in GCL at 24 weeks.

The study of the influence of sex on different pathologies is increasingly demanded. Our sex segregation analysis shows functional and structural changes with worse values in males, which coincides with previous studies and meta-analyses under the induction of glaucomatous damage [2, 63, 64, 67, 68]; but now we also show the differences with the proposed multiloaded neuroprotective treatment. Milestones that are not detected if this factor is not considered.

## Conclusions

In this work, the technological challenge of microencapsulating and subsequently releasing in a controlled manner of two molecules of low molecular weight and different solubility (dexamethasone and UDCA) in water, in combination with a neuroprotective protein (GDNF) for at least three months, has been successfully achieved. According to the mathematical model used in this work, it seems that the presence of dexamethasone does influence the distribution of UDCA in the microsphere matrix, increasing its initial release.Furthermore, the presence of UDCA did increase the dexamethasone release rate in the stage governed by the drug dissolution mechanism.

In addition, this study also presents the efficacy of this multitherapy formulation. It improved the functionality of RGCs with respect to untreated glaucomatous cohorts, and it achieved neuroretinal thicknesses and RGC counting similar to the healthy cohort, after a 6-month study. This co-loaded formulation could be an interesting therapy for ophthalmic degenerative pathologies such as glaucoma.

## Supplementary Information

Below is the link to the electronic supplementary material.

Supplementary file1 (DOCX 47 KB)

Supplementary file2 (DOCX 66 KB)

Supplementary file3 (DOCX 59 KB)

Supplementary file4 (DOCX 43 KB)

## Data Availability

The datasets generated during and/or analysed during the current study are available from the corresponding author on reasonable request.
