# Supplementary material for: Multi-loaded PLGA microspheres as neuroretinal therapy in a chronic glaucoma animal model
Source: Drug Deliv Transl Res. 2024 Oct 3;15(5):1660–84. doi: 10.1007/s13346-024-01702-x (PMC11968513; doi:10.1007/s13346-024-01702-x)
Supplement: Supplementary file 1 — Supplementary file1 (DOCX 47 KB) [file 13346_2024_1702_MOESM1_ESM.docx]

DRUG DELIVERY AND TRANSLATIONAL RESEARCH

MULTI-LOADED PLGA MICROSPHERES AS NEURORETINAL THERAPY IN A CHRONIC GLAUCOMA ANIMAL MODEL

Alba Aragón-Navas^1,2#^, MJ Rodrigo^3,4,5#^, Inés Munuera^4,5^, David García-Herranz^1,2^, Manuel Subías^4,5,6^, Pilar Villacampa^7^, Julián García-Feijoo^8,9^, Luis Pablo^3,4,5,6^, Elena Garcia-Martin^3,4,5^, Rocio Herrero-Vanrell^1,2,9,10^, Irene Bravo-Osuna^1,2,9,10*^.

Institutions:

^1^Innovation, Therapy and Pharmaceutical Development in Ophthalmology (InnOftal) Research Group, UCM 920415, Department of Pharmaceutics and Food Technology, Faculty of Pharmacy, Complutense University of Madrid, Madrid, Spain

^2^ Health Research Institute, San Carlos Clinical Hospital (IdISSC), Madrid, Spain

^3^ National Ocular Research Network RD21/0002/0050. RICORS Red de Enfermedades Inflamatorias (RD21/0002). Carlos III Health Institute, Spain

^4^ Department of Ophthalmology, Miguel Servet University Hospital, Zaragoza, Spain

^5^ Miguel Servet Ophthalmology Research Group (GIMSO), Aragon Health Research Institute (IIS Aragon), University of Zaragoza, Spain

^6^ Biotech Vision, Instituto Oftalmologico Quiron, Zaragoza, Spain

^7^ Department of Physiological Sciences, Faculty of Medicine and Health Sciences, University of Barcelona and Bellvitge Biomedical Research Institute (IDIBELL), Feixa Llarga s/n, 08907 l’Hospitalet de Llobregat, Spain.

^8^Department of Ophthalmology, San Carlos Clinical Hospital, Health Research Institute of the San Carlos Clinical Hospital (IdISSC), Madrid, Spain.

^9^ University Institute for Industrial Pharmacy (IUFI), School of Pharmacy, Complutense University of Madrid, Madrid, Spain

^10^ National Ocular Pathology Network (OFTARED), Carlos III Health Institute, Madrid, Spain

*Correspondence: [ibravo@ucm.es](mailto:ibravo@ucm.es)

#: equal contribution.

**Supplementary methods 2**

Anesthesia.

For corneal injections and intraocular pressure (IOP) measurements rats were previously sedated with a mixture of 3% sevoflurane gas and 1.5% oxygen. For ERG and OCT acquisitions general anesthesia by intraperitoneal injections of ketamine (60 mg/kg) and dexmedetomidine (0.25 mg/kg) was used, but also topical anesthesia with 1 mg/mL tetracaine + 4 mg/mL oxiburprocaine (Anestesico doble Colircusi®, Alcon Cusi® SA, Barcelona, Spain), and animal’s pupils were fully dilatated with tropicamide (10 mg/mL) and phenylephrine (100 mg/mL) (Alcon Cusi® SA, Barcelona, Spain).

Intraocular pressure (IOP).

IOP was assessed by a Tonolab^®^ tonometer (Tonolab, Tiolat Oy Helsinki, Finland). Six measurements were taken and averaged in each eye. Examinations were always done in all rats in the morning to avoid circadian fluctuation patterns, from both right and left eyes (always measuring right eye first) at baseline, every week during the first month, and at 6, 12, 18, and 24 weeks of follow-up study.

Electroretinography (ERG).

ERG (Roland consult RETIanimal^®^ ERG, Germany) was used to study neuroretinal functionality, measuring signal latency (in ms) and amplitude (in µV). Flash scotopic ERG and photopic negative responses (PhNR) protocols were performed. For scotopic ERG the animals were dark-adapted for 12 hours. Electrodes were placed as described: active ones on both right and left corneas, references on both body sides under the skin and the ground one near the tail. Acceptable impedance was considered if less than 2kW between electrodes. Both eyes were simultaneously tested using a Ganzfeld Q450 SC sphere stimulated by white LED flashes. There were performed 7 steps to analyze rod response (step 1: -40dB, 0.0003 cds/, 0.2 Hz [20 recordings averaged], step 2: -30dB, 0.003 cds/, 0.125 Hz [18 recordings averaged], step 3: -20dB, 0.03 cds/, 8.929 Hz [14 recordings averaged], step 4: -20dB, 0.03 cds/, 0.111 Hz [15 recordings averaged], step 5: -10dB, 0.3 cds/, 0.077 Hz [15 recordings averaged]), mixed rod-cone response (step 6: -40dB, 3.0 cds/, 0.067 Hz [12 recordings averaged], and oscillatory potentials (step 7: 0dB, 3.0 cds/, 29.412 Hz [10 recordings averaged]. PhNR test was performed after light adaptation to a blue background (470 nm, 25 cds/) stimulated with a red LED flash (625 nm, -10dB, 0.30 cds/, 1.199 Hz [20 recordings averaged]. Six animals (both sexes) were tested at baseline, 12 and 24 weeks.

Optical coherence tomography (OCT).

The neuroretinal structure was analyzed by OCT (Spectralis®, Heidelberg Engineering, Germany), quantifying retinal parameters thickness in micrometers (µm). Segmentation protocols were performed for the analysis of the Retina Posterior Pole (RPP), Ganglion Cell Layer (GCL), and peripapillary Retinal Nerve Fiber Layer (pRNFL). These protocols use 61 scans to analyze an area centered on the optic disc since these animals do not have macula. The RPP and GCL protocols analyze a 3 ${mm}^{2}$ area including 9 EDTRS (Early Disease Treatment Retinopathy Study) areas: a central ring (C) of 1 mm diameter, an inner ring of 2 mm diameter divided into inferior (II), superior (IS), nasal (IN) and temporal (IT) sectors and an outer ring of 3 mm diameter divided also into inferior (OI), superior (OS), nasal (ON) and temporal (OT) sectors. The pRNFL protocol analyzes 6 sectors: inferotemporal (IT), temporal (T), superotemporal (ST), superonasal (SN), nasal (N), and inferonasal (IN). A corneal-adapted contact lens power plane was used to acquire higher quality images. Measurements were taken in both eyes (always measuring the right eye first) at 0, 6, 12, 18, and 24 weeks. Six animals (both sexes) were tested at each time.
