# Supplementary material for: Multi-loaded PLGA microspheres as neuroretinal therapy in a chronic glaucoma animal model
Source: Drug Deliv Transl Res. 2024 Oct 3;15(5):1660–84. doi: 10.1007/s13346-024-01702-x (PMC11968513; doi:10.1007/s13346-024-01702-x)
Supplement: Supplementary file 2 — Supplementary file2 (DOCX 66 KB) [file 13346_2024_1702_MOESM2_ESM.docx]

DRUG DELIVERY AND TRANSLATIONAL RESEARCH

MULTI-LOADED PLGA MICROSPHERES AS NEURORETINAL THERAPY IN A CHRONIC GLAUCOMA ANIMAL MODEL

Alba Aragón-Navas^1,2#^, MJ Rodrigo^3,4,5#^, Inés Munuera^4,5^, David García-Herranz^1,2^, Manuel Subías^4,5,6^, Pilar Villacampa^7^, Julián García-Feijoo^8,9^, Luis Pablo^3,4,5,6^, Elena Garcia-Martin^3,4,5^, Rocio Herrero-Vanrell^1,2,9,10^, Irene Bravo-Osuna^1,2,9,10*^.

Institutions:

^1^Innovation, Therapy and Pharmaceutical Development in Ophthalmology (InnOftal) Research Group, UCM 920415, Department of Pharmaceutics and Food Technology, Faculty of Pharmacy, Complutense University of Madrid, Madrid, Spain

^2^ Health Research Institute, San Carlos Clinical Hospital (IdISSC), Madrid, Spain

^3^ National Ocular Research Network RD21/0002/0050. RICORS Red de Enfermedades Inflamatorias (RD21/0002). Carlos III Health Institute, Spain

^4^ Department of Ophthalmology, Miguel Servet University Hospital, Zaragoza, Spain

^5^ Miguel Servet Ophthalmology Research Group (GIMSO), Aragon Health Research Institute (IIS Aragon), University of Zaragoza, Spain

^6^ Biotech Vision, Instituto Oftalmologico Quiron, Zaragoza, Spain

^7^ Department of Physiological Sciences, Faculty of Medicine and Health Sciences, University of Barcelona and Bellvitge Biomedical Research Institute (IDIBELL), Feixa Llarga s/n, 08907 l’Hospitalet de Llobregat, Spain.

^8^Department of Ophthalmology, San Carlos Clinical Hospital, Health Research Institute of the San Carlos Clinical Hospital (IdISSC), Madrid, Spain.

^9^ University Institute for Industrial Pharmacy (IUFI), School of Pharmacy, Complutense University of Madrid, Madrid, Spain

^10^ National Ocular Pathology Network (OFTARED), Carlos III Health Institute, Madrid, Spain

*Correspondence: [ibravo@ucm.es](mailto:ibravo@ucm.es)

#: equal contribution.

**Supplementary methods**

With the purpose of analysing the possible mechanism involved in the drug release kinetics, the release experimental data were fitted in different kinetic models (zero and first order, Korsmeyer-Peppas, Hixson-Crowell, Higuchi, Baker-Londsdale, Weibull and Gallagher-Corrigan).

The *zero-order kinetic model* describes a pharmaceutical form whose area remains unchangeable through time with a constant and slow drug release where no equilibrium conditions are achieved. It is represented by the following equation:

$$Q=Q_{0}+K_{0}t$$

Where Q is the amount of released or dissolved drug, Q_0_ is the initial amount of drug in the dosage form, K_0_ is the zero-order constant, and t is the time [1].

The *first order kinetic model* corresponds to the general case in which the drug-liquid interface variation is directly proportional to the variation of the mass of remaining solid drug in the system, and therefore the release rate is proportional to that mass of remaining drug. It is described by this equation:

$$ln\left( \frac{Q_{t}}{Q_{0}} \right)= -Kt$$

Where, Q_t_ is the amount of drug released in a determined time, Q_0_ is the initial amount of drug in the solution and K is the first-order rate constant being its units time^-1^ [1].

The *Hixson-Crowell model* considers that the release rate is proportional to the mass of drug remaining to dissolve in the system raised to 2/3. This idea derives from considering the geometry of the solid particle to be dissolved and assumes that it is always spherical. Finally, the equation is expressed as follows:

$$M_{0}^{1/3}- M_{i}^{1/3}= Kt$$

Where M_0_ is the initial amount of drug, M_i_ is the remaining drug in the pharmaceutical dosage at a determined time, K is the constant and t the time [2].

The *Higuchi model* is based on Fick 1^st^ law. In this model, it is estimated that once the drug is dissolved, it must diffuse to leave the polymeric matrix formed by excipients. It is this diffusion, and not the dissolution of the drug, that controls its release.

Despite the simplicity of its equation, this model considers six hypotheses that need to be followed: (i) the initial drug concentration in the matrix is much higher than its solubility, (ii) the diffusion of the drug only occurs in one dimension, (iii) the particle size is much smaller than the thickness of the system, (iv) it is insignificant the swelling or dissolution behaviour of the system, (v) drugs diffuses in a constant manner, and (vi) perfect sink conditions in the release media.

$$Q = K\sqrt{t}$$

Where Q is the amount of drug release in an elapsed time (t) and K is the Higuchi release constant [3].

This Higuchi model has been the origin of many other release kinetic models that were appearing to explain the increasingly complex modified release systems, such as the *Korsmeyer-Peppas model* and the *Baker-Lonsdale model*.

In the case of Korsmeyer-Peppas model, not only Fickian diffusion through the polymeric matrix but also other phenomena such as matrix relaxation or erosion are considered.

$$\frac{M_{t}}{M_{0}}= Kt^{n}$$

Where M_t_/M_0_ represents the ratio of drug release at a determined time, K is the rate constant, t is the time and n is the release exponent. Only the first 60% drug release data points can be fitted into this equation. The exponent “n” indicates the drug mechanism of transport across the polymer [2]. These values change from thin films, cylinders, or spheres systems. In the case of spherical systems, if the n value is 0.43 the release suffers a Fickian diffusion – the drug is released through a diffusion due to a chemical potential gradient. If it is 0.85 is Case-II transport due to changes in the polymer such as relaxation and erosion. When an n value is between 0.43 and 0.85 is described as a non-Fickian release (anomalous), combining both phenomena [3].

The above-mentioned equation does not consider the burst effect, or the burst effect is zero. When it is not the case, this equation becomes [1]:

$$\frac{M_{t}}{M_{0}}= Kt^{n}+ b$$

The *Baker-Lonsdale model* explains the drug release from spherical matrixes, linearizing the data from microcapsules and microspheres. It follows the equation:

$$f_{t}= \frac{3}{2}\left[ 1- \left( 1-\frac{M_{t}}{M_{\infty}} \right)^{\frac{2}{3}} \right] - \frac{M_{t}}{M_{\infty}} = Kt$$

Where M_t_/$M_{\infty}$ is the fraction of drug released at a time (t) [1,2].

The *Weibull model* is an empirical equation used generally to linearize data, however, it has been adapted to the dissolution-release processes. The equation lacks kinetic fundaments and for this reason, it possesses deficiencies, and its use is controversial [1]. It is expressed as:

$$F= F_{max}\left( 1 - e^{-kt^{\beta}} \right)$$

Where F is the amount of drug dissolved in a determined time (t), $F_{max}$ is the total amount to be released, and k and β are constants. Although several criticisms, some attempts have been made in order to validate its use in release studies. Therefore, the β constant has been related to a different mechanism based on its values. In this way, if this constant has values lower than 0.75, it exists a Fickian diffusion. For values between 0.75 and 1.0, there is a combination of mechanisms between diffusion with another release mechanism. When β =1, it is compatible with the first-order model. Finally, when the value is above 1 (sigmoid curve), it is indicative of a complex mechanism of release [4].

Finally, the experimental data were fitted in the *Gallagher-Corrigan model* with some modifications. Gallagher and Corrigan developed a mathematical model that describes sigmoidal shape profiles, that usually occur in drug delivery systems where polymer undergoes degradation. The profile is described by the following equation that comprises the initial burst release because of the non-bounded drug to the matrix – the first addend –, followed by a slow-release due to matrix erosion, the second addend:

$$F_{TOT}= F_{B}\left( 1 - e^{-k_{B}t} \right)+ (1 -F_{B})\left( \frac{e^{kt-kT_{max}}}{1 +e^{kt-kT_{max}}} \right)$$

Where $F_{TOT}$ is the total fraction of drug release at a time (t), $F_{B}$ is the fraction of drug available for direct surface release (what they call “burst release”), $K_{B}$ is the release rate constant, and K and $T_{max}$ are the rate constant and the time to maximum drug release rate [5].

However, Gorrasi *et al* [6] modified this equation by adding a constant parameter (b) that considers the initial burst release. This factor shifts the model predictions up to fit the experimental release data. In the case that burst release does not exist, the b value is zero, becoming the original release equation (Gallagher-Corrigan equation). This equation is described as follows:

$$Y(t) = b + Y_{1}(1 - e^{{-k}_{1}t}) + Y_{2}\left( \frac{e^{{-k}_{2}(t_{2}- t)}}{1 + e^{{-k}_{2}(t_{2} - t)}} \right)$$

In this case, Y(t) is the drug fraction released in a t time, Y_1_ and Y_2_ are the relative amount of drug release in the first and second mechanisms, respectively; K_1_ and K_2_ are the kinetic constants of the first and second mechanisms, respectively; t_2_ is characteristic time of the second step mechanism and b is the burst parameter. This equation possesses 7 unknown parameters that were determined by adjusting the equation to the experimental data releases, using MATLAB® (MathWorks, USA).

**References**

[1] P. Costa, J.M. Sousa Lobo, Modeling and comparison of dissolution profiles, Eur J Pharm Sci. 13 (2001) 123–133. https://doi.org/10.1016/S0928-0987(01)00095-1.

[2] M. Padmaa Paarakh, P. Ani Jose, C.M. Setty, G.V.P. Christoper, RELEASE KINETICS-CONCEPTS AND APPLICATIONS, 12| International Journal of Pharmacy Research & Technology |. (n.d.).

[3] J. Siepmann, N.A. Peppas, Modeling of drug release from delivery systems based on hydroxypropyl methylcellulose (HPMC), Adv Drug Deliv Rev. 64 (2012) 163–174. https://doi.org/10.1016/J.ADDR.2012.09.028.

[4] V. Papadopoulou, K. Kosmidis, M. Vlachou, P. Macheras, On the use of the Weibull function for the discernment of drug release mechanisms, Int J Pharm. 309 (2006) 44–50. https://doi.org/10.1016/J.IJPHARM.2005.10.044.

[5] K.M. Gallagher, O.I. Corrigan, Mechanistic aspects of the release of levamisole hydrochloride from biodegradable polymers, J Control Release. 69 (2000) 261–272. https://doi.org/10.1016/S0168-3659(00)00305-9.

[6] G. Gorrasi, G. Attanasio, L. Izzo, A. Sorrentino, Controlled release mechanisms of sodium benzoate from a biodegradable polymer and halloysite nanotube composite, Polym Int. 66 (2017) 690–698. https://doi.org/10.1002/PI.5309.
