# Supplementary material for: Multi-loaded PLGA microspheres as neuroretinal therapy in a chronic glaucoma animal model
Source: Drug Deliv Transl Res. 2024 Oct 3;15(5):1660–84. doi: 10.1007/s13346-024-01702-x (PMC11968513; doi:10.1007/s13346-024-01702-x)
Supplement: Supplementary file 3 — Supplementary file3 (DOCX 59 KB) [file 13346_2024_1702_MOESM3_ESM.docx]

DRUG DELIVERY AND TRANSLATIONAL RESEARCH

MULTI-LOADED PLGA MICROSPHERES AS NEURORETINAL THERAPY IN A CHRONIC GLAUCOMA ANIMAL MODEL

Alba Aragón-Navas^1,2#^, MJ Rodrigo^3,4,5#^, Inés Munuera^4,5^, David García-Herranz^1,2^, Manuel Subías^4,5,6^, Pilar Villacampa^7^, Julián García-Feijoo^8,9^, Luis Pablo^3,4,5,6^, Elena Garcia-Martin^3,4,5^, Rocio Herrero-Vanrell^1,2,9,10^, Irene Bravo-Osuna^1,2,9,10*^.

Institutions:

^1^Innovation, Therapy and Pharmaceutical Development in Ophthalmology (InnOftal) Research Group, UCM 920415, Department of Pharmaceutics and Food Technology, Faculty of Pharmacy, Complutense University of Madrid, Madrid, Spain

^2^ Health Research Institute, San Carlos Clinical Hospital (IdISSC), Madrid, Spain

^3^ National Ocular Research Network RD21/0002/0050. RICORS Red de Enfermedades Inflamatorias (RD21/0002). Carlos III Health Institute, Spain

^4^ Department of Ophthalmology, Miguel Servet University Hospital, Zaragoza, Spain

^5^ Miguel Servet Ophthalmology Research Group (GIMSO), Aragon Health Research Institute (IIS Aragon), University of Zaragoza, Spain

^6^ Biotech Vision, Instituto Oftalmologico Quiron, Zaragoza, Spain

^7^ Department of Physiological Sciences, Faculty of Medicine and Health Sciences, University of Barcelona and Bellvitge Biomedical Research Institute (IDIBELL), Feixa Llarga s/n, 08907 l’Hospitalet de Llobregat, Spain.

^8^Department of Ophthalmology, San Carlos Clinical Hospital, Health Research Institute of the San Carlos Clinical Hospital (IdISSC), Madrid, Spain.

^9^ University Institute for Industrial Pharmacy (IUFI), School of Pharmacy, Complutense University of Madrid, Madrid, Spain

^10^ National Ocular Pathology Network (OFTARED), Carlos III Health Institute, Madrid, Spain

*Correspondence: [ibravo@ucm.es](mailto:ibravo@ucm.es)

#: equal contribution.

**Supplementary results**

Supplementary Table: Electroretinography (ERG). Comparison of electroretinography response in the four cohorts. Only the parameters with statistical significance were filled. Notice that the amplitude of bipolar cells with the lightest stimuli (b wave step 1 in μV), followed by photoreceptors (a wave step 2 in μV) and retinal ganglion cells (PhNR in μV) found more differences. Subanalysis by eye laterality and by sex in each cohort. R=right eye; L=left eye; M=male; F=female; w=weeks; G: Glaucoma cohort; IV: intravitreal injection; NP: neuroprotective; DX: dexamethasone; UDCA: ursodeoxycholic acid; GDNF: glial derived neurotrophic factor.

| **ERG PARAMETERS** | | | **12 w** | | | | | | | | | **24 w** | | | | | | | | |
| --- | --- | --- | --- | --- | --- | --- | --- | --- | --- | --- | --- | --- | --- | --- | --- | --- | --- | --- | --- | --- |
|  |  |  | **HEALTHY** | | **G** | | **G+ IV non-loaded** | | **G+IV NP (Dx+ UDCA+ GDNF)** | | **Sig. ANOVA** | **HEALTHY** | | **G** | | **G+ IV non-loaded** | | **G + IV NP (Dx+ UDCA+ GDNF)** | | **Sig. ANOVA** |
|  |  |  | **R vs L** | **M vs F** | **R vs L** | **M vs F** | **R vs L** | **M vs F** | **R vs L** | **M vs F** |  | **R vs L** | **M vs F** | **R vs L** | **M vs F** | **R vs L** | **M vs F** | **R vs L** | **M vs F** |  |
| **ROD response** | **Step 1**  -40 dB  0.0003 cds/m^2^  0.2 Hz  [20 recordings average] | **a_[ms]** |  |  |  |  |  |  |  |  |  |  |  |  |  |  |  |  |  |  |
|  |  | **b_[ms]** |  |  |  |  |  |  |  |  | 0.001^a=0.003, e=0.018^ |  |  |  |  |  |  |  |  |  |
|  |  | **a-wave_[μV]** |  |  |  |  |  |  |  |  |  |  |  |  |  | 15.34±12.21 vs 52.47±19.46 |  | 14.27±10.61 vs 64.46±42.75 |  |  |
|  |  | **b-wave_[μV]** |  |  | 67.50±29.39 vs 114.75±36.35 |  | 50.44 1±5.87 vs 131.98±64.24 |  |  |  |  |  |  | 44.65±30.84 vs 139.67±78.16 | 20.87 ±1.48 vs 68.43 ±26.05 |  |  | 36.02±11.63 vs 96.52±41.59 |  |  |
|  | **Step 2**  -30 dB  0.003 cds/m^2^  0.125 Hz  [18 recordings average] | **a_[ms]** |  |  |  |  |  |  |  |  |  |  |  |  |  |  |  |  |  |  |
|  |  | **b_[ms]** |  |  |  |  |  |  |  |  |  |  |  |  |  |  |  | 69.86±2.34 vs 66.46±2.27 |  |  |
|  |  | **a-wave_[μV]** | 49.96±24.65vs 12.31±6.57 |  |  | 348.00 ±56.93 vs 560.00 ±176.12 | 10.32±10.28 vs 39.08 ± 20.54 |  |  |  | 0.038^b=0.041^ |  |  |  |  | 3.64±0.35 vs 60.73±28.96 |  | 14.91±10.08 vs 51.32±39.20 |  |  |
|  |  | **b-wave_[μV]** |  |  |  |  |  |  |  |  |  |  |  | 343.33±105.42 vs 514.67±163.23 |  |  |  |  |  |  |
|  | **Step 3**  -20 dB  0.03 cds/m^2^  8.929 Hz  [14 recordings average] | **a_[ms]** |  |  |  |  |  |  |  |  |  |  |  |  |  |  |  |  |  |  |
|  |  | **b_[ms]** |  |  |  |  |  |  |  |  | <0.001^a<0.001, b=0.004, c=0.001^ |  |  |  |  |  |  |  |  | 0.037 |
|  |  | **a-wave_[μV]** |  |  |  |  |  |  | 50.89±44.81 vs 117.92±71.91 |  |  |  |  |  |  |  |  | 40.88±13.98 vs 89.40±32.76 |  |  |
|  |  | **b-wave_[μV]** |  |  |  |  |  |  |  |  |  |  |  |  |  |  |  |  |  |  |
|  | **Step 4**  -20 dB  0.03 cds/m^2^  0.111 Hz  [15 recordings average] | **a_[ms]** |  |  |  |  |  |  |  |  |  |  |  |  |  |  |  |  |  |  |
|  |  | **b_[ms]** |  |  |  |  |  |  |  |  | 0.028^b=0.034^ |  |  |  |  |  |  |  |  | 0.003 ^a=0.007, e=0.025^ |
|  |  | **a-wave_[μV]** |  |  |  |  |  |  |  |  | 0.007^a=0.021, b=0.021^ |  |  |  |  |  |  |  |  |  |
|  |  | **b-wave_[μV]** |  |  |  | 447.00 ±79.57 vs 703.33 ±185.26 |  |  |  |  |  |  |  |  |  |  |  |  |  |  |
|  | **Step 5**  -10 dB  0.3 cds/m^2^  0.077 Hz  [15 recordings average] | **a_[ms]** |  |  |  |  |  |  |  |  |  |  |  |  |  |  |  |  |  |  |
|  |  | **b_[ms]** |  |  |  |  |  |  |  |  | 0.004^a=0.006, b=0.034, c=0.045^ |  |  |  |  |  |  |  |  | 0.021^a=0.023^ |
|  |  | **a-wave_[μV]** |  |  |  | 123.63 ±21.88 vs 220.00 ±75.35 |  |  |  |  |  |  |  |  |  |  |  |  |  |  |
|  |  | **b-wave_[μV]** |  |  |  |  |  |  |  |  |  |  |  |  |  |  |  |  |  |  |
| **MIX ROD-CONE response** | **Step 6**  0 dB  3.0 cds/m^2^  0.067 Hz  [12 recordings average] | **a_[ms]** |  |  |  |  |  |  |  |  | 0.024 |  |  |  |  |  |  |  |  | 0.047 |
|  |  | **b_[ms]** |  |  |  |  |  |  |  |  | 0.011^b=0.016^ |  |  |  |  |  |  |  |  |  |
|  |  | **a-wave_[μV]** |  |  |  |  |  |  |  |  |  |  |  |  |  | 281.67±108.52 vs 106.27±32.22 |  |  |  |  |
|  |  | **b-wave_[μV]** |  |  |  |  |  |  |  |  |  |  |  |  |  |  |  |  |  |  |
| **OSCILLATORY POTENTIALS** | **Step 7**  0 dB  3.0 cds/m^2^  29.412 Hz  [10 recordings average) | **a_[ms]** |  |  |  |  |  |  |  |  |  |  |  |  |  |  |  |  |  |  |
|  |  | **b_[ms]** |  |  |  |  |  |  |  |  |  |  |  |  |  |  |  |  |  |  |
|  |  | **a-wave_[μV]** |  |  |  |  | 25.76±25.14 vs 98.62±45.16 |  |  |  |  | 21.18±24.60 vs 61.20±33.91 |  |  |  |  |  |  |  |  |
|  |  | **b-wave_[μV]** |  |  |  | 29.13 ±25.67 vs 105.47 ±35.02 | 57.78±30.09 vs 151.44±85.03 |  |  |  |  |  |  |  |  |  |  |  |  |  |
| **PhNR** | | **a_[ms]** |  |  |  |  |  |  |  |  |  |  |  |  |  |  |  |  |  |  |
|  |  | **b_[ms]** |  |  |  |  |  |  |  |  |  |  |  |  |  |  |  |  |  |  |
|  |  | **PhNR_[ms]** |  |  |  |  |  |  |  |  |  |  |  |  |  |  |  |  |  |  |
|  |  | **a-wave_[μV]** |  |  |  |  |  |  |  |  |  |  |  |  |  |  |  |  |  |  |
|  |  | **b-wave_[μV]** |  |  |  |  |  |  |  |  |  | 24.85±9.05 vs 64.97±31.43 |  |  |  | 24.37±15.60 vs 81.22 5±4.93 |  | 34.48±8.93 vs 83.58±30.6 |  |  |
|  |  | **PhNR_[μV]** |  |  | 22.45±13.42 vs 47.85±17.69 | 19.24 ±15.47 vs 25.67 ±13.41 |  |  |  |  |  |  |  | 17.83 1±7.66 vs 36.52±31.19 | 4.23 ±1.97 vs 26.89 ±17.71 |  |  |  |  |  |
