# Supplementary material for: Multi-loaded PLGA microspheres as neuroretinal therapy in a chronic glaucoma animal model
Source: Drug Deliv Transl Res. 2024 Oct 3;15(5):1660–84. doi: 10.1007/s13346-024-01702-x (PMC11968513; doi:10.1007/s13346-024-01702-x)
Supplement: Supplementary file 4 — Supplementary file4 (DOCX 43 KB) [file 13346_2024_1702_MOESM4_ESM.docx]

DRUG DELIVERY AND TRANSLATIONAL RESEARCH

MULTI-LOADED PLGA MICROSPHERES AS NEURORETINAL THERAPY IN A CHRONIC GLAUCOMA ANIMAL MODEL

Alba Aragón-Navas^1,2#^, MJ Rodrigo^3,4,5#^, Inés Munuera^4,5^, David García-Herranz^1,2^, Manuel Subías^4,5,6^, Pilar Villacampa^7^, Julián García-Feijoo^8,9^, Luis Pablo^3,4,5,6^, Elena Garcia-Martin^3,4,5^, Rocio Herrero-Vanrell^1,2,9,10^, Irene Bravo-Osuna^1,2,9,10*^.

Institutions:

^1^Innovation, Therapy and Pharmaceutical Development in Ophthalmology (InnOftal) Research Group, UCM 920415, Department of Pharmaceutics and Food Technology, Faculty of Pharmacy, Complutense University of Madrid, Madrid, Spain

^2^ Health Research Institute, San Carlos Clinical Hospital (IdISSC), Madrid, Spain

^3^ National Ocular Research Network RD21/0002/0050. RICORS Red de Enfermedades Inflamatorias (RD21/0002). Carlos III Health Institute, Spain

^4^ Department of Ophthalmology, Miguel Servet University Hospital, Zaragoza, Spain

^5^ Miguel Servet Ophthalmology Research Group (GIMSO), Aragon Health Research Institute (IIS Aragon), University of Zaragoza, Spain

^6^ Biotech Vision, Instituto Oftalmologico Quiron, Zaragoza, Spain

^7^ Department of Physiological Sciences, Faculty of Medicine and Health Sciences, University of Barcelona and Bellvitge Biomedical Research Institute (IDIBELL), Feixa Llarga s/n, 08907 l’Hospitalet de Llobregat, Spain.

^8^Department of Ophthalmology, San Carlos Clinical Hospital, Health Research Institute of the San Carlos Clinical Hospital (IdISSC), Madrid, Spain.

^9^ University Institute for Industrial Pharmacy (IUFI), School of Pharmacy, Complutense University of Madrid, Madrid, Spain

^10^ National Ocular Pathology Network (OFTARED), Carlos III Health Institute, Madrid, Spain

*Correspondence: [ibravo@ucm.es](mailto:ibravo@ucm.es)

#: equal contribution.

Supplementary Table 1 – Fitting values of constants (K) and statistical parameters (R^2^, R^2^_adjusted_ and SSE) in the different kinetics models from UDCA release.

| ***Models***  **Steps (from day X to day X)** | **UDCA release** | | | | | | | | | | | |
| --- | --- | --- | --- | --- | --- | --- | --- | --- | --- | --- | --- | --- |
|  | **F-UDCA** | | | | **F** | | | | **F+GDNF** | | | |
|  | **K** | **R^2^** | **R^2^_adjusted_** | **SSE** | **K** | **R^2^** | **R^2^_adjusted_** | **SSE** | **K** | **R^2^** | **R^2^_adjusted_** | **SSE** |
| *Zero-order* |  |  |  |  |  |  |  |  |  |  |  |  |
| 1-21 | 2.591 | 0.905 | 0.858 | 0.138 | 0.256 | 0.820 | 0.730 | 3.237 | 0.396 | 0.923 | 0.884 | 2.945 |
|  |  |  |  |  |  |  |  |  |  |  |  |  |
| 21-42 | 2.378 | 0.952 | 0.927 | 70.530 | 1.138 | 0.949 | 0.924 | 17.050 | 2.357 | **0.970** | 0.955 | 42.320 |
| 42-70 | 0.123 | 0.825 | 0.782 | 2.748 | 0.033 | 0.748 | 0.664 | 0.177 | 0.073 | 0.728 | 0.638 | 0.967 |
| *First order* |  |  |  |  |  |  |  |  |  |  |  |  |
| 1-21 | -0.022 | 0.928 | 0.892 | 0.008 | -0.032^a^ | 0.837^a^ | 0.674^a^ | 0.017^a^ | -0.065 | **0.971** | 0.965 | 0.168 |
| 21-42 | -0.123 | 0.952 | 0.927 | 0.188 | -0.041^b^ | 0.939^b^ | 0.919^b^ | 0.053^b^ |  |  |  |  |
| 42-70 | -0.003 | 0.881 | 0.842 | 0.001 | -0.0003 | 0.960 | 0.940 | 1.2e-06 | -0.0004 | 0.766 | 0.649 | 1.47e-05 |
| *Korsmeyer-Peppas* | |  |  |  |  |  |  |  |  |  |  |  |
| 1-14 | 0.061 | 0.642 | 0.284 | 0.008 | 0.162^c^ | **0.987**^c^ | 0.980^c^ | 0.002^c^ | 0.160 | 0.848 | 0.696 | 0.017 |
| 14-28 | 1.616 | 0.824 | 0.649 | 0.135 |  |  |  |  | 1.599 | 0.950 | 0.901 | 0.032 |
| Hixson-Crowell | |  |  |  |  |  |  |  |  |  |  |  |
| 1-21 | -0.002 | 0.904 | 0.856 | 8.03e-05 | -0.008 | 0.828 | 0.742 | 0.003 | -0.011 | 0.914 | 0.872 | 0.002 |
| 21-49 | -0.068 | 0.928 | 0.916 | 0.726 | -0.038 | 0.904 | 0.872 | 0.075 | -0.227 | 0.923 | 0.898 | 2.102 |
| 49-70 |  |  |  |  | -0.00089 | 0.907 | 0.860 | 1.995e-05 | -0.002 | 0.793 | 0.690 | 0.0003 |
| *Higuchi* |  |  |  |  |  |  |  |  |  |  |  |  |
| 7-21 | 0.013 | 0.947 | 0.894 | 1.796e-05 | 0.031^c^ | 0.935^c^ | 0.903^c^ | 0.0005^c^ | 0.067 | 0.919 | 0.838 | 0.00074 |
| 21-42 | 0.471 | 0.940 | 0.911 | 0.028 | 0.255 | 0.957 | 0.936 | 0.006 | 0.496 | **0.972** | 0.959 | 0.014 |
| 42-70 | 0.042 | 0.914 | 0.885 | 0.0004 | 0.010 | 0.778 | 0.703 | 6.343e-05 | 0.021 | 0.760 | 0.680 | 0.0003 |
| *Baker-Lonsdale* | |  |  |  |  |  |  |  |  |  |  |  |
| 1-21 | 3.102e-05 | 0.878 | 0.816 | 3.017e-08 | 0.0005 | 0.867 | 0.801 | 8.102e-06 | 0.0005 | 0.849 | 0.773 | 1.029e-05 |
| 21-49 | 0.014 | 0.935 | 0.914 | 0.007 | 0.005 | 0.915 | 0.886 | 0.001 | 0.028^d^ | 0.836^d^ | 0.671^d^ | 0.015^d^ |
| 49-70 | 0.005 | 0.950 | 0.924 | 0.0003 | 0.0002 | 0.907 | 0.861 | 7.313e-07 |  |  |  |  |
| *Weibull* |  |  |  |  |  |  |  |  |  |  |  |  |
| 7-21 | 0.358 | 0.937 | 0.873 | 0.005 | 0.184^c^ | **0.988**^c^ | 0.982^c^ | 0.002^c^ | 0.708 | 0.924 | 0.848 | 0.025 |
| 21-42 | 0.191 | **0.977** | 0.966 | 0.006 | 2.113 | 0.968 | 0.952 | 0.040 | 4.912 | 0.939 | 0.878 | 0.205 |
| 42-70 | 1.51 | **0.989** | 0.985 | 0.004 | 0.109 | 0.809 | 0.745 | 0.0005 |  |  |  |  |
| *Gallagher-Corrigan* | K1= 0.025  K2= 0.396 | **0.9985** | 0.9976 | 0.0032 | K1 = 1.117  K2= 0.339 | **0.9996** | 0.9994 | 0.000026 | K1 = 1.256  K2= 0.265 | **0.9994** | 0.9990 | 0.0017 |
| *Gallagher-Corrigan + Gorrasi correction* | K1= 0.009  K2= 0.376 | **0.9998** | 0.9994 | 0.0007 | K1= 0.303  K2= 0.350 | **0.9998** | 0.9996 | 0.00014 | K1= 0.047  K2= 0.300 | **0.9998** | 0.9997 | 4.76e-04 |

^a^From day 1 to 14.^b^ From day 14 to 42. ^c^ From day 1 to 21.^d^ Until day 35. K in Korsmeyer-Peppas model refers to n. R^2^$\geq$0.97 in bold.

Supplementary Table 2 - Fitting values of constants (K) and statistical parameters (R^2^, R^2^_adjusted_ and SSE) in the different kinetics models from DX release.

| ***Models***  **Steps (from day X to day X)** | **DX release** | | | | | | | | | | | |
| --- | --- | --- | --- | --- | --- | --- | --- | --- | --- | --- | --- | --- |
|  | **F-DX** | | | | **F** | | | | **F+GDNF** | | | |
|  | **K** | **R^2^** | **R^2^_adjusted_** | **SSE** | **K** | **R^2^** | **R^2^_adjusted_** | **SSE** | **K** | **R^2^** | **R^2^_adjusted_** | **SSE** |
| *Zero-order* |  |  |  |  |  |  |  |  |  |  |  |  |
| 1-28 | 0.212 | 0.920 | 0.893 | 1.806 | 0.646 | 0.780 | 0.707 | 54.450 | 2.256 | **0.993** | 0.986 | 3.080 |
|  |  |  |  |  |  |  |  |  | 0.316 | 0.893 | 0.787 | 1.170 |
| 28-70 | 1.707 | **0.985** | 0.982 | 60.84 | 0.773 | 0.929 | 0.915 | 62.680 | 0.912 | **0.974** | 0.970 | 30.120 |
| *First order* |  |  |  |  |  |  |  |  |  |  |  |  |
| 1-28 | -0.027 | 0.848 | 0.797 | 0.062 | -0.009^a^ | 0.965^a^ | 0.948^a^ | 0.0008^a^ | -0.056^b^ | 0.857^b^ | 0.786^b^ | 0.115^b^ |
| 28-56 | -0.060 | **0.994** | 0.992 | 0.011 | -0.020 | **0.993** | 0.991 | 0.001 | -0.015^c^ | **0.972**^c^ | 0.965^c^ | 0.006^c^ |
| 56-70 | -0.019 | 0.919 | 0.838 | 0.003 | -0.002 | 0.848 | 0.696 | 5.36e-05 | -0.005 | 0.925 | 0.851 | 0.0002 |
| *Korsmeyer-Peppas* | |  |  |  |  |  |  |  |  |  |  |  |
| 1-28 | 0.238 | **0.984** | 0.979 | 0.007 | 0.222 | **0.977** | 0.969 | 0.008 | 0.358 | **0.986** | 0.983 | 0.015 |
| 28-42 | 2.426 | **0.998** | 0.998 | 0.003 | 0.716 | **0.973** | 0.947 | 0.001 |  |  |  |  |
| 42-63 |  |  |  |  |  |  |  |  |  |  |  |  |
| *Hixson-Crowell* | |  |  |  |  |  |  |  |  |  |  |  |
| 1-28 | -0.003 | 0.923 | 0.897 | 0.0004 | -0.002 | 0.945 | 0.939 | 0.001 | -0.003 | 0.951 | 0.946 | 0.003 |
| 28-70 | -0.039 | **0.976** | 0.971 | 0.053 |  |  |  |  |  |  |  |  |
| *Higuchi* |  |  |  |  |  |  |  |  |  |  |  |  |
| 1-28 | 0.013 | **0.973** | 0.964 | 5.745e+05 | 0.047 | 0.916 | 0.887 | 0.002 | 0.083 | 0.951 | 0.934 | 0.004 |
| 28-70 | 0.225 | **0.978** | 0.974 | 0.008 | 0.112 | 0.951 | 0.941 | 0.005 | 0.124 | **0.984** | 0.981 | 0.002 |
| *Baker-Lonsdale* | |  |  |  |  |  |  |  |  |  |  |  |
| 1-28 | 5.667E-05 | 0.961 | 0.947 | 6.117E-08 | 0.0009 | 0.865 | 0.820 | 5.407e-05 | 0.002 | 0.906 | 0.875 | 0.0002 |
| 28-70 | 0.004 | 0.919 | 0.902 | 0.002 | 0.003 | 0.946 | 0.935 | 0.0006 | 0.004 | **0.982** | 0.978 | 0.0005 |
| *Weibull* |  |  |  |  |  |  |  |  |  |  |  |  |
| 1-21 | 0.233 | **0.991** | 0.987 | 0.003 | 0.270 | **0.980** | 0.970 | 0.008 | 0.457 | **0.990** | 0.986 | 0.011 |
| 21-70 | 2.432 | 0.964 | 0.958 | 0.270 | 0.938 | 0.952 | 0.944 | 0.054 | 0.971 | 0.935 | 0.924 | 0.079 |
| *Gallagher-Corrigan* | K1= 0.006  K2= 0.184 | **0.9936** | 0.9899 | 0.0056 | K1=0.747  K2= 0.159 | **0.9966** | 0.9953 | 0.0012 | K1=0.163  K2= 0.164 | **0.986** | 0.977 | 0.008 |
| *Gallagher-Corrigan + Gorrasi correction* | K1= 0.409  K2= 0.134 | **0.9994** | 0.9990 | 4.999e-04 | K1= 0.228  K2=0.166 | **0.9996** | 0.9992 | 1.491e-04 | K1 = 0.116  K2= 0.154 | **0.997** | 0.996 | 0.001 |

^a^From day 7 to 28. ^b^From day 1 to 21. ^c^From day 21 to 56. K in Korsmeyer-Peppas model refers to n. R^2^$\geq$0.97 in bold.

Supplementary Table 3 - Fitting values of constants (K) and statistical parameters (R^2^, R^2^_adjusted_ and SSE) in the different kinetics models from GDNF release.

| ***Models***  **Steps (from day X to day X)** | **GDNF release** | | | |
| --- | --- | --- | --- | --- |
|  | **F+GDNF** | | | |
|  | **K** | **R^2^** | **R^2^_adjusted_** | **SSE** |
| *Zero-order* |  |  |  |  |
| 1-28 | 85.110 | 0.863 | 0.818 | 5.308e+05 |
|  |  |  |  |  |
| 28-49 | 8.789 | 0.963 | 0.944 | 733.100 |
| 49-98 | 83.520 | **0.973** | 0.969 | 3.991e+05 |
| *First-order* |  |  |  |  |
| 1-28 | -0.013 | 0.829 | 0.772 | 0.017 |
| 28-49 | -0.001 | 0.963 | 0.944 | 1.235e-05 |
| 49-90 | -0.009 | **0.978** | 0.974 | 0.002 |
| *Korsmeyer-Peppas* | |  |  |  |
| 1-28 | 0.118 | **0.997** | 0.996 | 2.920e-04 |
| 28-49 | 0.043 | 0.957 | 0.936 | 1.429e-05 |
| 49-84 | 0.508 | 0.954 | 0.943 | 0.003 |
| *Hixson-Crowell* | |  |  |  |
| 1-28 | -0.055 | 0.875 | 0.834 | 0.200 |
| 28-49 | -0.006 | 0.963 | 0.944 | 0.00036 |
| 49-90 | -0.063 | 0.946 | 0.935 | 0.315 |
| *Higuchi* |  |  |  |  |
| 1-28 | 0.032 | 0.968 | 0.957 | 3.802e-04 |
| 28-49 | 0.006 | 0.961 | 0.941 | 2.335e-06 |
| 49-90 | 0.073 | 0.942 | 0.931 | 0.002 |
| *Baker-Lonsdale* | |  |  |  |
| 1-28 | 0.0007 | 0.905 | 0.874 | 2.651e-05 |
| 28-49 | 9.738e-05 | 0.963 | 0.944 | 9.063e-08 |
| 49-90 | 1.545e-05 | **0.993** | 0.991 | 1.650e-05 |
| *Weibull* |  |  |  |  |
| 1-28 | 0.147 | **0.997** | 0.995 | 5.469e-04 |
| 28-49 | 0.057 | 0.957 | 0.935 | 2.56e-05 |
| 49-90 | 0.848 | 0.931 | 0.917 | 0.016 |
| *Gallagher-Corrigan* | K1= 1.269  K2=0.070 | **0.993** | 0.992 | 0.003 |
| Gallagher-Corrigan + Gorrasi correction | K1= 0.119  K2= 0.089 | **0.9988** | 0.9985 | 5.431e-04 |

K in Korsmeyer-Peppas model refers to n. R^2^$\geq$0.97 in bold.
